# Distinct immunometabolic signatures in peripheral blood mononuclear cells are linked to systemic inflammation in type 2 diabetes and diabetic kidney disease

**DOI:** 10.1186/s43556-026-00523-3

**Published:** 2026-08-08

**Authors:** Clara Luna-Marco, José F. Català-Senent, Julia Cacace, Alberto Hermo-Argibay, Omar A. Hernández-López, María Pelechá-Salvador, Jonay Pantoja, Asunción Sancho, Jose M. Vila, Carlos Morillas, Celia Bañuls, Milagros Rocha, Víctor M. Víctor, Susana Rovira-Llopis

**Affiliations:** 1 Department of Physiology, Faculty of Medicine, University of Valencia, INCLIVA (Biomedical Research Institute Valencia), Valencia, Spain; 2 Microbiology and Ecology Department, University of Valencia, Valencia, Spain; 3 Service of Endocrinology and Nutrition, University Hospital Doctor Peset, Foundation for the Promotion of Health and Biomedical Research in the Valencian Region (FISABIO), MITOMETS, Valencia, Spain; 4 Service of Nephrology, University Hospital Doctor Peset, Foundation for the Promotion of Health and Biomedical Research in the Valencian Region (FISABIO), Valencia, Spain

**Keywords:** Type 2 diabetes, Diabetic kidney disease, Immunometabolism, Peripheral blood mononuclear cells (PBMCs), Transcriptomic profiling, Inflammation

## Abstract

Diabetic kidney disease (DKD) is a frequent complication of type 2 diabetes and is closely linked to systemic inflammation. Peripheral blood mononuclear cells (PBMCs) are markers of systemic inflammatory and metabolic stress. It is unknown if metabolism-related transcriptomic alterations in these cells is associated with DKD. Using the nCounter® Human Metabolic Pathways Panel we profiled PBMC metabolic transcripts in individuals with type 2 diabetes or DKD and in controls (*n* = 12/group), and integrated transcriptomic data with clinical, inflammatory, and mitochondrial parameters. Patients with DKD showed increased inflammatory biomarkers and reduced PBMC mitochondrial membrane potential and mass, consistent with mitochondrial dysfunction. Metabolism-related transcriptomic profiling identified 13 differentially expressed genes across groups. DKD subjects displayed downregulation of *SLC7A11* versus controls and *HLA-DQA1* versus type 2 diabetes, and upregulation of *CPT1A* and *GBA1* versus controls. *CPT1A* upregulation was confirmed by RT-qPCR and supported by external GEO datasets, though ROC analyses indicated a limited discriminatory performance. Pathway analyses revealed enrichment of immune-related, fatty acid oxidation, and fructose-6-phosphate pathways and reduced cell proliferation pathways in DKD patients. Inflammatory markers correlated positively with energy-regulating pathways and negatively with anabolic processes. In conclusion, these findings suggest that PBMCs reflect immunometabolic remodeling in response to DKD, thus highlighting an association between systemic inflammation, mitochondrial dysfunction, and altered energy metabolism in circulating immune cells.

**Supplementary Information:**

The online version contains supplementary material available at 10.1186/s43556-026-00523-3.

## Introduction

Chronic kidney disease (CKD) affects one in seven adults in Spain [1] and is particularly prevalent in subjects with type 2 diabetes [2, 3], mainly due to poor glycaemic control. Indeed, approximately 20%–40% of people with type 2 diabetes develop diabetic kidney disease (DKD) [4], which markedly increases cardiovascular risk and health care costs [3, 5]. Unfortunately, there is no cure for DKD, and treatment is limited to blood pressure and glycaemic control through diet or drugs [6]. Furthermore, most biomarkers of DKD, such as glomerular filtration rate and albuminuria, are detected once the disease has developed and damage is irreversible [7].

Immunometabolism refers to how alterations in the metabolic pathways of immune cells regulate their activity and shape immune responses. Among the different immune cell types, peripheral blood mononuclear cells (PBMCs) are involved in pathogen detection, immune activation, and coordination of both innate and adaptive immunity [8]. Interestingly, mitochondrial dysfunction in PBMCs is known to be a hallmark of type 2 diabetes and is worsened by poor glycaemic control [9]. These cells act as frontline metabolic and stress sensors, and so their gene expression is a useful indicator of overall metabolic and immune system status. For these reasons, and because they can be obtained by minimally invasive means, PBMCs are widely used in clinical and translational research as surrogate tissue to monitor disease states, therapeutic responses and overall health. In this context, novel technologies are available, such as NanoString nCounter® system [10], a molecular characterization platform that enables reproducible, accurate and multiplexed quantification of mRNAs. In contrast to large-scale 'omics'-based approaches, this technology can be applied to low sample numbers and requires minimal processing, thereby facilitating its use in validation studies and consolidating its clinical usefulness.

Alterations in blood cell counts have been reported in patients with CKD and correlate with the progression of the disease [11, 12]. Specifically, patients with CKD are characterized by increased numbers of the intermediate monocyte subpopulation, which is in turn associated with undermined renal function [13, 14]. Interestingly, higher monocyte-to-lymphocyte ratio (MLR) is positively associated with the risk of all-cause and cardiovascular mortality in patients with CKD, supporting its potential as a predictive biomarker of kidney injury and mortality risk [15]. Although circulating immune cell counts are reliable and accessible biomarkers of DKD, they do not provide information concerning the molecular pathways underlying immune dysregulation, for which analysis of intrinsic gene expression profiles in immune cells is useful. This specific molecular signature may be related to immune cell infiltration into the diabetic kidney, a pathological mechanism reported to be central to the progression of DKD [16, 17]; therefore, it might predict disease onset in patients with type 2 diabetes. However, how DKD progression is driven by the intrinsic molecular alterations known to occur in PBMCs during the disease has not yet been determined.

In this study, we investigated the transcriptomic profiles of PBMCs from patients with type 2 diabetes –with and without DKD– with respect to control subjects, and their association with inflammatory and metabolic parameters. Across the groups, 13 differentially expressed genes were found, among which *CPT1A, GBA1, SLC7A11 and HLA-DQA1* stood out as the most deregulated. *CPT1A* was upregulated, though it showed limited discriminatory performance in the ROC analysis and functional analysis did not reveal any dependance of PBMCs on CPT1A function. Pathway enrichment analysis reflected a shift toward energy generation and compromised growth, biosynthesis, and long-term metabolic control. Altogether, our results suggest that metabolism-related PBMC transcriptomic signatures are linked to systemic inflammation in overweight individuals with type 2 diabetes, alone and with DKD, which reflects the coordinated immunometabolism changes in key energy-regulating pathways that take place alongside immune activation.

## Results

### Type 2 diabetes is characterised by metabolic alterations, whereas DKD is associated with heightened inflammation

Anthropometrical measurements and blood analyses were performed to characterize the different groups (see Table 1). Subjects with type 2 diabetes – with or without DKD (groups DKD and D, respectively) – were slightly older than controls (group C) and the group with type 2 diabetes had higher body-mass index (BMI) than the other groups. Thus, clinical variables were compared after adjusting for age and BMI. Following adjustment, glucose levels, HOMA-IR index, Systemic Inflammatory Index (SII) and MLR no longer showed statistically significant differences among groups. Nevertheless, as expected, both groups with type 2 diabetes maintained higher levels of glycated haemoglobin (HbA1c) than controls (*p* < 0.05), and DKD group presented higher albumin/creatinine ratio, leukocyte and neutrophil counts and neutrophil-to-lymphocyte ratio (NLR) than controls and subjects with type 2 diabetes, indicating greater systemic inflammation. Treatments and proportion of patients in the KDIGO categories are reported in Tables S1 and S2, respectively. In short, groups with type 2 diabetes presented metabolic alterations in line with the disease, regardless of age and BMI adjustments, and the DKD group showed greater inflammation compared to controls and the other type 2 diabetes patients.

**Table 1:** Characteristics of the study population

	C​	D​	DKD​	*p* value​	Age & BMI adjusted* p*-value​
n​	12​	12​	12​	-​	-​
Sex (% women)​	66.7%​	41.7%​	50%​	ns​	-​
Age (years)​	49 ± 8​	58 ± 11*​	61 ± 7**​	< 0.01​	-​
BMI (kg/m^2^)​	24.7 ± 3.2​	29.5 ± 4.7* ​	26.1 ± 4.0​	< 0.05 ​	-​
HbA1c (DCCT %​)	5.30 (5.04–5.50)​	7.14 (6.08–7.84)***​	6.70 (6.36–7.24)***​	< 0.001​	< 0.05​
Glucose (mg/dL)​	88 (82.5–95.25)​	115.5 (92.5–167)**​	123 (95.25–143.5)**​	< 0.01​	ns​
Insulin (uUI/mL)​	6.60 (4.25–10.03)​	10.35 (4.50–20.65)​	11.20 (7.40–17.43)*​	ns​	ns​
HOMA-IR​	1.425 (0.87–2.027)​	3.134 (1.831–6.5)*​	3.807 (2.285–5.215)**​	< 0.01​	ns​
Cholesterol (mg/dL)​	208 (178–255)​	160.5 (122–198)**​	141 (28–153)**​	< 0.01​	< 0.05​
HDL (mg/dL)​	61.33 ± 12.6 ​	50.0 ± 11.2*​	52.4 ± 12.5​	ns​	ns​
LDL (mg/dL)​	133.6 ± 37.1​	81.0 ± 49.0**​	64.8 ± 21.0***​	< 0.001​	< 0.01​
Triglycerides (mg/dL)​	81.0 (52.3–136.3)​	86.5 (74.5–160.5)​	125.0 (63.3–194.5)​	ns​	ns​
Atherogenic Index of Plasma​	0.15 ± 0.34​	0.36 ± 0.33​	0.36 ± 0.4​	ns​	ns​
Glomerular FiltrationRate (ml/min/1.73m^2^)​	99.5 ± 12.2​	91.58 ± 18.02​	86.42 ± 23.385​	ns​	ns​
Albumin/creatinine ratio (mg/g)​	5.00 (4.00–7.00)​	8.50 (4.45–12.75)​	42.50 (35.00–88.75)***^†††​^	< 0.001​	< 0.01​
High sensitivity C-Reactive Protein (mg/L)​	0.95 (0.42–2.17)​	1.32 (0.623–3.56)​	0.91 (0.52–9.92)​	ns​	ns​
Plasmatic Homocystein(µmol/L)​	9.78 ± 2.99​	10.30 ± 2.94​	13.15 ± 4.29​	ns​	ns​
Leukocytes (×10^9^/L)​	6.42 ± 0.92​	7.10 ± 1.49​	8.95 ± 2.25***^††^​	< 0.01​	< 0.01​
Neutrophils (×10^9^/L)​	3.73 ± 0.89​	3.78 ± 1.16​	5.91 ± 1.79**^†††^​	< 0.001​	< 0.01​
Lymphocytes (×10^9^/L)​	1.91 ± 0.45​	2.51 ± 0.73​	2.14 ± 0.67​	ns​	ns​
Monocytes (×10^9^/L)​	0.50 (0.40–0.68)​	0.50 (0.40–0.60)​	0.55 (0.50–0.68)​	ns​	ns​
Eosinophils (×10^9^/L)​	0.2 (0.12–0.30)​	0.15 (0.10–0.40)​	0.15 (0.10–0.20)​	ns​	ns​
Platelets (×10^9^/L)​	250 ± 59​	220 ± 43​	228 ± 85​	ns​	ns​
NLR​	2.06 ± 0.70​	1.63 ± 0.76​	2.97 ± 1.16*^†††^​	< 0.01​	< 0.05​
PLR​	138.8 ± 53.4​	94.4 ± 30.3*​	118.8 ± 67.5​	ns​	ns​
SII​	531 ± 263​	356 ± 169​	716 ± 457^††^​	< 0.05​	ns​
MLR​	0.27 (0.23–0.33)​	0.20 (0.17–0.24)*​	0.31 (0.25–0.38)^††^​	< 0.05​	ns​

*C* control subjects, *D* patients with type 2 diabetes, *DKD* patients with type 2 diabetes and diabetic kidney disease, *BMI* body-mass index, *HbA1c* glycated haemoglobin, *HOMA-IR* Homeostatic Model Assessment for Insulin Resistance, *HDL* high density lipoprotein, *LDL* low density lipoprotein, *NLR* neutrophil-to-lymphocyte ratio, *PLR* platelet-to-lymphocyte ratio, *SII* Systemic Inflammatory Index, *MLR* monocyte-to-lymphocyte ratio. Normally distributed parameters are shown as mean ± SD, while non-normally distributed data are expressed as medians (25th-75th quantiles). Comparisons between groups were performed using an ANOVA and Fisher LSD or Kruskal-Wallis and Dunn tests for normally and non-normally distributed data, respectively. The χ^2^ test was used to compare proportions. The influence of age and BMI was tested and corrected using a general linear model. ns = non-significant, *p < 0.05, **p < 0.01, ***p < 0.001 vs. C; †*p* < 0.05, ††*p* < 0.01, †††*p* < 0.001 vs. D

### DKD is associated with PBMC mitochondrial dysfunction and enhanced neutrophil–endothelium interactions

**Fig. 1:**
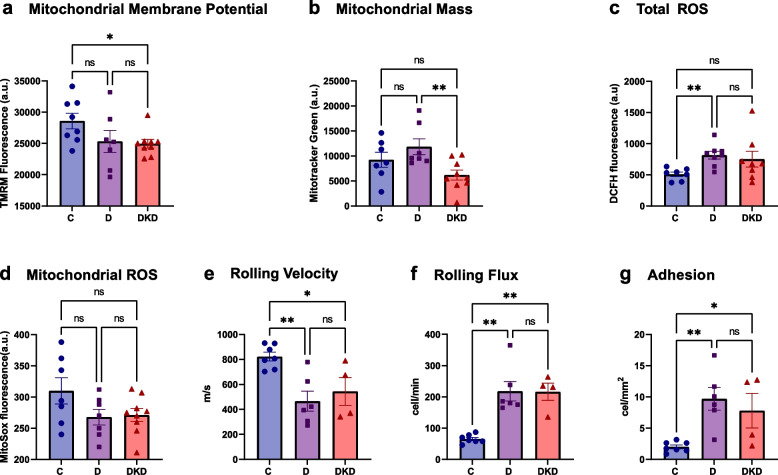
Mitochondrial membrane potential (**a**), mitochondrial mass (**b**), total (**c**) and mitochondrial reactive oxygen species (ROS) (**d**) in peripheral blood mononuclear cells (PBMCs) measured by flow cytometry, and neutrophil-endothelium interactions: Rolling velocity (**e**), rolling flux (**f**) and adhesion (**g**) measured by a parallel flow-chamber system. One way ANOVA, ns = non-significant, *p < 0.05, **p < 0.01. TMRM: Tetramethyl rhodamine methyl ester, DCFH-DA: 2',7'-dichlorodihydrofluorescein diacetate, C = control subjects, D = patients with type 2 diabetes, DKD = patients with type 2 diabetes and diabetic kidney disease

We characterized the PBMC phenotype in terms of mitochondrial function by measuring mitochondrial mass, membrane potential, and reactive oxygen species (ROS) production. Tetramethyl rhodamine methyl ester (TMRM) fluorescence was lower in DKD vs controls, indicating a lower mitochondrial membrane potential (Fig. 1a). Mitochondrial mass, measured by Mitotracker Green, was specifically reduced in DKD compared with overweight subjects with type 2 diabetes (Fig. 1b). Interestingly, total ROS production (2',7'-dichlorodihydrofluorescein diacetate fluorescence – DCFH-DA) was higher in overweight subjects with type 2 diabetes vs controls and showed a tendency towards higher levels in DKD vs controls (*p* = 0.06) (Fig. 1c), whereas mitochondrial ROS did not differ among groups (MitoSox, Fig. 1d).

Given the alterations in systemic inflammatory markers in the type 2 diabetes and DKD groups, we evaluated neutrophil–endothelium interactions as a surrogate marker of subclinical atherosclerosis. This analysis was performed to assess whether the observed inflammatory profile corresponded with a functionally altered leukocyte phenotype that might confer an increased risk of cardiovascular disease. Velocity was lower (Fig. 1e), while rolling flux and adhesion were higher (Fig. 1f-g) in both groups with type 2 diabetes, regardless of the presence or absence of DKD.

These results demonstrate that, despite a lack of differences in ROS production in our DKD group, there was a decrease in mitochondrial mass and membrane potential, thus suggesting mitochondrial dysfunction. Moreover, type 2 diabetes in general was associated with increased neutrophil–endothelial interactions, a marker of subclinical atherosclerosis.

### Type 2 diabetes and DKD possess distinct metabolism-related PBMC transcriptomic signatures

**Fig. 2:**
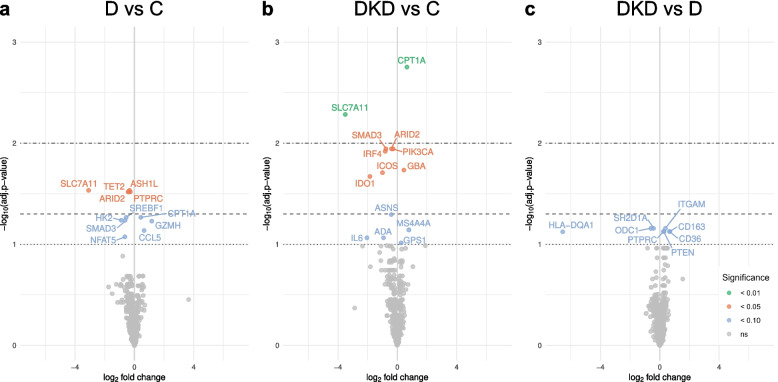
Differentially expressed genes represented in volcano plots: D versus C comparison (**a**), DKD versus C comparison (**b**), and DKD versus D comparison (**c**). Green dots represent significantly altered genes with -log10(*p* value) < 0.01, orange dots represent significantly changed genes with -log10(*p* value) < 0.05, blue dots represent those with -log10(*p* value) < 0.1 and grey dots represent those that are not statistically significant (ns). C= Control, D= Type 2 diabetes, DKD= Diabetic kidney disease

To define the transcriptomic profile of the PBMCs from our cohort revealed by the Nanostring analysis, we detected differentially expressed genes (DEGs) with two-by-two comparisons. We assumed that differences in cell type proportions among groups were unlikely to influence gene expression, since monocyte and lymphocyte counts (Table 1) and markers for the most abundant PBMC cell types (T cells, B cells, and NK cells) did not differ among groups (Fig. S1). DEGs are represented in the volcano plots of Fig. 2. The type 2 diabetes group showed downregulation of solute carrier family 7 member 11 (*SLC7A11*), ASH1 like histone lysine methyltransferase (*ASH1L*), AT-rich interaction domain 2 (*ARID2*), tet methylcytosine dioxygenase 2 (*TET2*) and protein tyrosine phosphatase receptor type C (*PTPRC*) compared to controls (*p* < 0.05; Fig. 2a). Patients with DKD presented underexpression of *SLC7A11* (*p* < 0.01), indoleamine 2,3-dioxygenase 1 (*IDO1*)*,* inducible T cell costimulator (*ICOS*)*,* interferon regulatory factor 4 (*IRF4*)*,* SMAD family member 3 (*SMAD3*)*, ARID2* and phosphatidylinositol-4,5-bisphosphate 3-kinase catalytic subunit alpha (*PIK3CA*) (*p* < 0.05) and overexpression of carnitine palmitoyl transferase 1A* (CPT1A)* (*p* < 0.01) and glucosylceramidase beta 1 (*GBA1)* (*p* < 0.05) compared to controls (Fig. 2b). When DKD and type 2 diabetes groups were compared, no significant changes were found (Fig. 2c). However, major histocompatibility complex class II DQ alpha 1 (*HLA-DQA1*) exhibited a markedly negative-fold change, thus indicating notable downregulation. In total, 13 genes were differentially expressed among groups; of these, we would highlight *CPT1A*, *GBA1*, *SLC7A11* and *HLA-DQA1* due to their highly differential expression and/or pronounced fold-change.

### Enriched pathways in PBMCs reflect the systemic metabolic and inflammatory alterations of type 2 diabetes and DKD

**Fig. 3:**
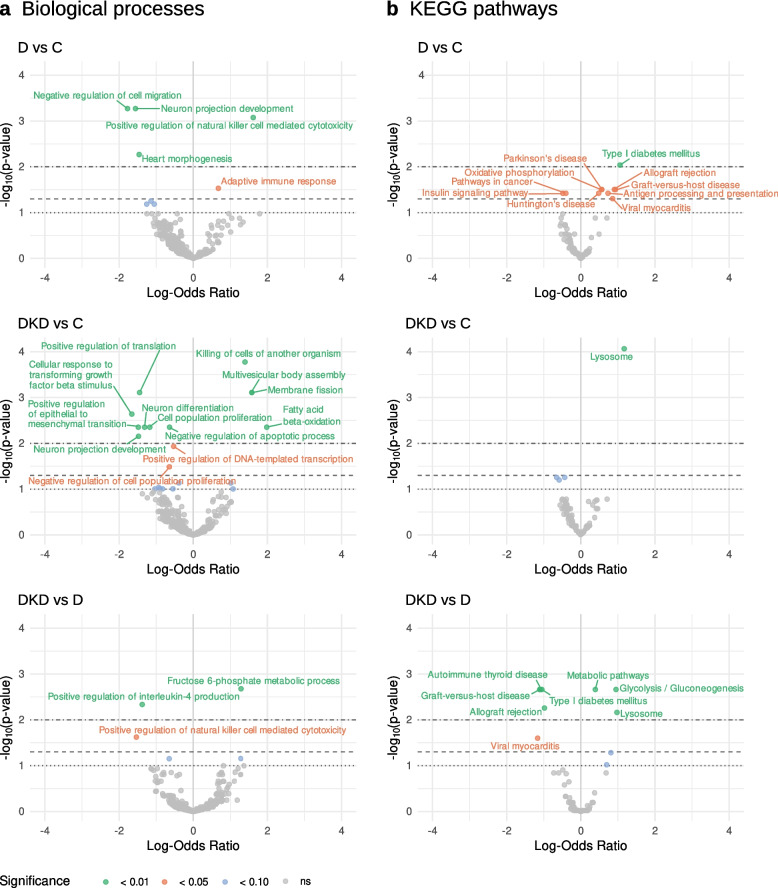
Pathway enrichment of Gene Ontology (GO) biological processes (**a**) and the Kyoto Encyclopedia of Genes and Genomes (KEGG) pathways (**b**). Green dots represent significantly altered pathways with *p* value < 0.01, orange dots are those significantly changed pathways with *p* value < 0.05, blue dots represent those with *p* value < 0.1 and grey dots represent those that are not statistically significant (ns). C=Control, D= Type 2 diabetes, DKD= Diabetic kidney disease

After identifying DEGs among groups, we carried out a functional analysis of gene ontology (GO) biological processes (Fig. 3a) and Kyoto Encyclopedia of Genes and Genomes (KEGG) pathways (Fig. 3b) in order to identify under- or overexpressed functions.

Immune-related pathways –“positive regulation of natural killer cell mediated cytotoxicity” (*p* < 0.01) and “adaptive immune response” (*p* < 0.05)– were enriched in overweight subjects with type 2 diabetes compared to controls, while “multivesicular body assembly”, “membrane fission” and “fatty acid beta-oxidation" were more pronounced in DKD vs controls (*p* < 0.01; Fig. 3a). However, only the “fructose 6-phosphate pathway” and “receptor-mediated endocytosis” were enriched in the DKD groups when compared with the type 2 diabetes group (*p* < 0.01). In contrast, pathways that were downregulated in type 2 diabetes vs controls included those involved in cellular dynamics –“negative regulation of cell migration”, “neuron projection development” and “heart morphogenesis” (*p* < 0.01)– while pathways that were downregulated in DKD vs controls included those related with immunity and cellular dynamics; namely, “positive regulation of translation”, “cellular response to transforming growth factor beta stimulus”, “neuron differentiation”, “negative regulation of apoptotic process”, “positive regulation of epithelial to mesenchymal transition”, “cell population proliferation”, “neuron projection development” (*p* < 0.01) and “negative regulation of cell population proliferation” (*p* < 0.05). Lastly, “positive regulation of interleukin-4 production” (*p* < 0.01) and “positive regulation of natural killer cell-mediated cytotoxicity” (*p* < 0.05) were downregulated in DKD vs the type 2 diabetes patients.

Regarding KEGG pathway enrichment, overweight subjects with type 2 diabetes showed overrepresentation of genes involved in “type I diabetes mellitus” (*p* < 0.01; Fig. 3b), “oxidative phosphorylation”, “Parkinson’s disease” and “Huntington's disease” and some immune-related pathways –“graft-versus-host disease”, “allograft rejection”, “antigen processing and presentation” and “viral myocarditis” (*p* < 0.05)– and underrepresentation of the “insulin signalling pathway” and “pathways in cancer” (*p* < 0.05). When DKD were compared with controls, only the “lysosome” pathway was upregulated in the former (*p* < 0.01). Finally, metabolism-related pathways such as “glycolysis/gluconeogenesis” and “metabolic pathways” were upregulated in DKD vs type 2 diabetes (*p* < 0.01) and immune-related pathways such as “graft-versus-host disease” and “autoimmune thyroid disease” were downregulated in DKD vs type 2 diabetes (*p* < 0.01).

In short, GO and KEGG analyses confirmed the enrichment of multiple metabolic and inflammatory pathways in the type 2 diabetes and DKD groups, indicating that PBMCs reflect the systemic metabolic and inflammatory alterations observed in these patients.

### Inflammation markers are associated with upregulation of catabolic pathways and downregulation of anabolic pathways

Individual pathway scores, which summarize the combined expression patterns of genes within a pathway, were calculated to identify similar patterns across pathways. Pathway scores that followed the same pattern of expression in the three study groups were categorized in five differentiated clusters (Fig. S2). Importantly, we observed one clear and consistent trend; namely, that one group of pathways (Cluster 4) presented similar downregulation in both type 2 diabetes and DKD patients when compared with controls (Fig. S2d). Cluster 4 included amino acid synthesis and transporters, reactive oxygen response and transcriptional regulation. Interestingly, fatty acid oxidation was the pathway that marked the greatest distinction in patients with DKD, in which it was specifically upregulated (Fig. S2e). Notably, it did not follow any clear or consistent pattern shared by other pathways, appearing to be distinct and uniquely regulated in this condition.

**Fig. 4:**
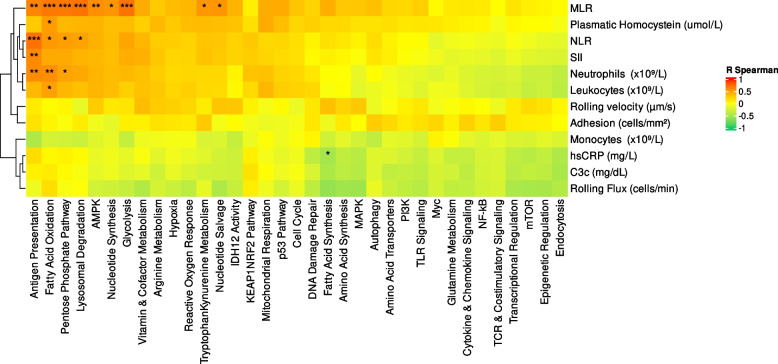
Inflammation correlation matrix. Spearman correlation between pathway scores and inflammation-related variables shown in a colour gradient from red (positive) to green (negative). Pathways and variables were clustered using the Euclidean distance along with the complete linkage method. C3c = C3 complement; hsCRP = high sensitivity C-Reactive Protein; NLR = neutrophil-to-lymphocyte ratio; SII = systemic inflammatory index; MLR = monocyte-to-lymphocyte ratio; *p < 0.05, **p < 0.01, ***p < 0.001

Next, we aimed to evaluate whether coordinated changes in biological pathway activity were associated with our subjects’ metabolic and inflammatory status. Thus, bivariate correlations between pathway scores and individuals' metabolism-related variables and inflammation-related variables were assessed. After multiple-testing correction, none of the individual metabolic parameters listed in Table 1 showed significant correlations with the pathway scores. In contrast, strong correlations were observed between systemic inflammation markers (also included in Table 1) and a group of metabolism-related pathways expressed in PBMCs (Fig. 4). SII, MLR, and NLR, which are composite blood cell count-derived indices that reflect systemic inflammation and immune status, were positively correlated with antigen presentation, thus reflecting immune activation and the engagement of adaptive immune responses. Together with plasmatic homocysteine, immune cell counts and derived indices –especially MLR– were positively correlated with fatty acid oxidation and other metabolic and energy-regulating pathways, including the pentose phosphate pathway, lysosomal degradation, AMPK, nucleotide synthesis and glycolysis. These associations point to coordinated immunometabolic remodelling of fundamental energy-regulating pathways implicated in immune activation. Also of interest, high-sensitivity C-reactive protein (hsCRP), another inflammatory biomarker, correlated negatively with fatty acid synthesis, a classic anabolic pathway. Considered together, these observations demonstrate that systemic inflammation markers correlate positively with fundamental energy-regulating pathways and negatively with anabolic processes.

### CPT1A upregulation marks DKD-associated PBMC metabolic remodelling with limited discriminatory performance and preserved fatty acid oxidation

To investigate whether the expression changes observed using NanoString were reproducible across independent cohorts, we compared our results (GSE316126) with publicly available transcriptomic datasets of similar characteristics in the Gene Expression Omnibus (GEO) repository. The selected transcriptomic datasets included data from PBMCs of subjects with type 2 diabetes and DKD, subjects with type 2 diabetes without DKD, and healthy control samples, (GSE142153, GSE154881 and GSE185011). Four genes were selected for validation based on: 1) DKD-specific upregulation (*CPT1A* and *GBA1*); 2) their being affected by type 2 diabetes independently of DKD presence (*SLC7A11*); and 3) their being distinctive in type 2 diabetes versus DKD (*HLA-DQA1*). Compared to the other two datasets, the GSE142153 dataset had the greater number of subjects with DKD [23] and additionally included a group of subjects with end-stage renal disease (ESRD). Interestingly, of the genes we found to be upregulated in our cohort, *CPT1A* was consistently upregulated in subjects with DKD versus their respective controls in both GSE142153 and GSE154881 datasets (Fig. S3a and b). On the other hand, *GBA1* was upregulated in DKD samples in the GSE154881 dataset (Fig. S3b) and in subjects with ESRD in the GSE142153 dataset, though not in DKD in the latter dataset (Fig. S3a). The results for the genes that were downregulated in our cohort –*SLC7A11* and *HLA-DQA1*– did not align with the findings of the datasets. None of the genes were differentially expressed among groups in the GSE185011 dataset (Fig. S3c). Heatmaps summarizing the different dataset findings regarding the selected genes are shown in Fig. S3d.

**Fig. 5:**
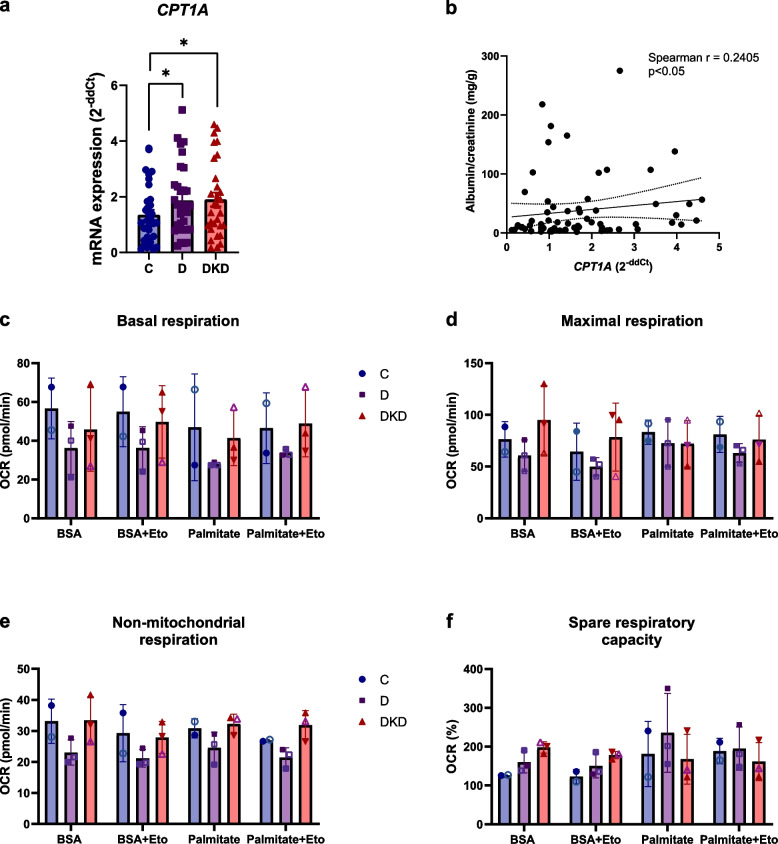
Validation of *CPT1A* expression in a separate cohort (**a**) and correlation of *CPT1A* expression with albumin/creatinine ratio (**b**) (total *n* = 95). CPT1A activity in peripheral blood mononuclear cells (PBMCs) from control subjects (C, *n* = 2), patients with type 2 diabetes (D, *n* = 3), and DKD patients (DKD, *n* = 3), measured with the Palmitate Oxidation Stress Test. Oxygen consumption rate (OCR) was evaluated as basal respiration (**c**), maximal respiration (**d**), non-mitochondrial respiration (**e**) and spare respiratory capacity (**f**). *p < 0.05; Eto = etomoxir, C = control subjects, D = patients with type 2 diabetes, DKD = patients with type 2 diabetes and diabetic kidney disease

Overall, *CPT1A* upregulation was the most consistent change detected, especially in the GSE142153 dataset, which closely resembled our cohort in ethnicity and diagnostic criteria. Therefore, and to put to the test our results obtained via the transcriptomic analysis with Nanodrop, we assessed the expression of *CPT1A* at mRNA level with quantitative reverse transcription polymerase chain reaction (RT-qPCR) and observed that both type 2 diabetes and DKD groups displayed an upregulation with respect to controls (*p* < 0.05; Fig. 5a). Moreover, in the same patients, *CPT1A –*increased expression showed a moderate positive correlation with albumin/creatinine ratio (Spearman r = 0.241, *p* < 0.05; Fig. 5b). The characteristics of the participants in the validation cohort are summarized in Table S3.

Lastly, in order to study the potential role of *CPT1A* as a biomarker for DKD, we carried out a diagnostic performance analysis in the Nanostring and RT-qPCR validation cohorts. *CPT1A* was evaluated as a discriminatory biomarker across the three group comparisons: controls vs. DKD patients (C vs. DKD), patients with type 2 diabetes vs patients with DKD (D vs. DKD), and both groups combined vs. DKD (C + D vs. DKD). As shown in Table S4 and Fig. S4, *CPT1A* reflected a strong discriminatory performance in the NanoString cohort, particularly for controls vs. DKD (AUC = 0.903, power = 0.991) and C + D vs. DKD (AUC = 0.785, power = 0.837), exceeding the conventional 0.80 power threshold in both cases. The type 2 diabetes vs. DKD comparison performed more modestly (AUC = 0.667, power = 0.300), which is a reflection of the biological challenge of separating two similar pathologies. In contrast, the performance in the RT-qPCR validation cohort was substantially poorer across comparisons. Given that the cohorts were broadly comparable (Table S3), this variability is probably attributable to methodological differences; i.e., amplification-based versus amplification-free approaches.

In order to provide a functional validation of the differential expression of *CPT1A* in type 2 diabetes and DKD groups, we performed a Seahorse-based palmitate oxidation test (Fig. 5c-f). The characteristics of the participants included in the functional validation are summarized in Table S5. Although preliminary, our data revealed no significant differences between groups in basal or maximal mitochondrial respiration; likewise, no differences were observed in non-mitochondrial respiration or spare respiratory capacity. Neither palmitate supplementation nor CPT1A inhibition with etomoxir significantly altered mitochondrial respiratory parameters. Overall, these results did not help to confirm whether, or to what extent, PBMCs rely on fatty acid oxidation.

### Diabetes- and DKD-derived PBMCs alter kidney cell receptor expression for PBMC-enriched ligands

Using the Cellinker platform, we further explored PBMC-kidney crosstalk by searching the kidney expression levels of receptors for ligands that were differentially expressed among study groups in our PBMC dataset [18]. We detected several ligands in the DEG list whose corresponding receptors are highly expressed in the kidney (Fig. S5a), and several receptors in the DEG list whose corresponding ligands are highly expressed in the kidney (Fig. S5b).

**Fig. 6:**
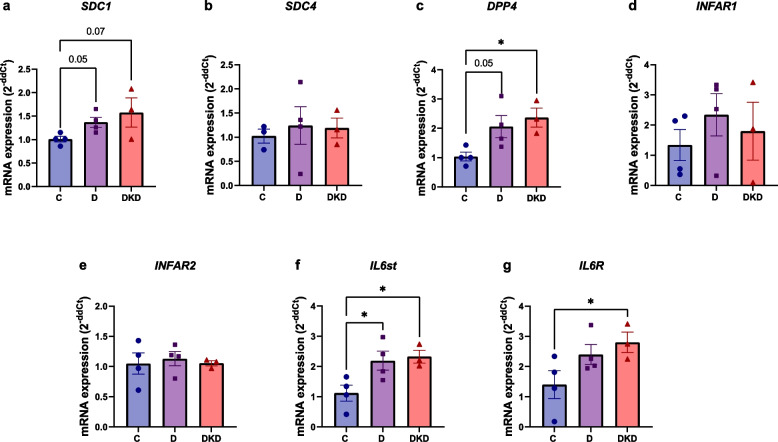
Gene expression (mRNA) of kidney receptors for differentially expressed genes in peripheral blood mononuclear cells (PBMCs): *SDC1* and *SDC4* (**a**-**b**, receptors for CCL5), *DPP4* (**c**, receptor for PTPRC), *INFAR1* and *2* (**d**-**e**, receptors for PIK3CA) and *IL6st* and *IL6R* (**f**-**g**, receptors for IL6). *p < 0.05; C = control subjects, D = patients with type 2 diabetes, DKD = patients with type 2 diabetes and diabetic kidney disease

As a preliminary approach to characterize these potential interactions between PBMC ligands and kidney receptors, we incubated PBMCs from healthy subjects and patients with type 2 diabetes with or without DKD (*n* = 3–4/group) with HEK293 cells, a human embryonic kidney-derived cell line (Fig. 6). The characteristics of the participants included in this part of the study are summarized in Table S5. Several receptors (Syndecan 1 and 4 (*SDC1*, *SDC4*), Dipeptidyl Peptidase 4 (*DPP4*), IL6 receptors (*IL6st*, *IL6R*), Interferon Alpha and Beta Receptor Subunit 1 and 2 (*INFAR1* and *INFAR2*) were selected based on their high expression levels in the kidney, with at least one representative receptor included for each DEG ligand present in PBMCs. After 24 h incubation, *SDC1* showed a slight increase in mRNA expression in kidney cells incubated with PBMCs from type 2 diabetes and DKD groups compared to controls (Fig. 6a; *p* = 0.05 and *p* = 0.07, respectively) following the upregulation of C–C Motif Chemokine Ligand (*CCL5*) gene expression in PBMCs of type 2 diabetes patients vs controls, as revealed by Nanostring. No changes were found for *SDC4* (Fig. 6b). *DPP4* was upregulated in kidney cells cultured with PBMCs from the DKD and type 2 diabetes groups vs controls (Fig. 6c; *p* < 0.05), which may have been a response to upregulation of *CCL5*, or a compensatory upregulation to lower expression of its other ligands (*PTPRC* and *PIK3CA*) in PBMCs. No changes were found in *INFAR1* or *2* (Fig. 6d-e), whereas *IL6st* and *IL6R* displayed higher expression in kidney cells cultured with PBMCs from DKD and type 2 diabetes patients vs controls (Fig. 6f-g; *p* < 0.05), when expression of *IL6* was downregulated in DKD vs controls.

These results point to alterations, either by positive regulation or compensatory mechanisms, in the transcriptional expression of receptors that are highly expressed in kidney cells. These alterations may be a response to PBMC-derived DEGs, thereby supporting the possibility of intercellular crosstalk.

## Discussion

Immunometabolism is a growing area of research within the field of type 2 diabetes, due to its demonstrated role in glycaemic control, insulin resistance and inflammation. Despite significant advances, how immunometabolism influences the onset, pathophysiology or progression of DKD is less understood. In this study, we have used NanoString technology to examine the transcriptomic profiles of PBMCs and thus explore the mRNA expression of metabolism-related genes and its relationship with clinical parameters and inflammation in overweight patients with type 2 diabetes and in patients with DKD.

Mitochondrial dysfunction and oxidative stress are key features of metabolic diseases, contributing to impaired energy metabolism, cellular damage, and disease progression. Herein, we verify that mitochondrial dysfunction is a hallmark of PBMCs from subjects with DKD.

Two DEGs were significantly overexpressed in DKD vs controls: *CPT1A* and *GBA1*. Importantly, comparisons with other available datasets supported upregulation of *CPT1A*, but not of *GBA1*, in DKD. In addition, RT-qPCR revealed that our validation cohort also presented higher expression of *CPT1A* in DKD vs controls, which was associated with higher albumin/creatinine ratio. CPT1A is a key enzyme in the mitochondrial oxidation of long-chain fatty acids [19], and its expression in PBMCs has been associated with adiposity, as enhanced levels are present in obesity. This supports the role of PBMCs as “sentinel” cells capable of reflecting gene expression profiles of internal tissues, such as adipose tissue [20]. Accordingly, in the present study, the BMI of the diabetic cohort fell within the overweight – obese range (29 ± 5 kg/m^2^), and there was a tendency towards increased *CPT1A* in the type 2 diabetes group versus controls. Interestingly, *CPT1A* expression was markedly higher in subjects with DKD, who had similar BMI to controls, which points to a different mechanism for the induction of *CPT1A* expression during kidney damage in the absence of adiposity.

In silico validation indicated that *CPT1A* upregulation was especially noticeable in the GSE142153 cohort, which had a similar ethnic background and diagnosis criteria [21] to those of our cohort, and that *CPT1A* was consistently higher in patients with DKD or ESRD (this was also the case in the GSE154881 dataset) [22]. In the GSE185011 dataset on the other hand, the expression of *CPT1A* was similar among control, type 2 diabetes and DKD groups, perhaps due to differences in DKD disease stage and ethnicity [23]. Although RT-qPCR data were consistent with our transcriptomic results, ROC and AUC analyses revealed a strong discriminatory performance of *CPT1A* between control subjects and DKD patients in the NanoString cohort, whereas the performance in the RT-qPCR cohort was more modest. Regarding functional validation with the Palmitate Oxidation Stress Test, we could not define whether or to what extent PBMCs relied on fatty acid oxidation in our cohorts. Although the data are preliminary and limited by a small sample size, the Seahorse-based assessment of palmitate oxidation provided a complementary functional perspective supporting the transcriptional observations regarding CPT1A, and should therefore be interpreted as exploratory evidence. Overall, we consider that our associations are biologically valid at the mRNA level, although further validation in independent, larger cohorts is required to confirm their robustness.

Pathway analysis revealed that our subjects with type 2 diabetes displayed an activation of both innate and adaptive immune responses that was not evident in controls. In our subjects with DKD, metabolic-related pathways (such as fatty acid beta-oxidation) rather than immunity-related pathways were upregulated, together with multivesicular body assembly and membrane fission pathways. Interestingly, genes involved in the negative regulation of apoptosis were downregulated in DKD vs controls, indicating an enhanced induction of programmed cell death in blood cells during DKD. In fact, different regulated cell death mechanisms are now recognized as key contributors to the pathogenesis of kidney diseases [24].

Some of the differences we have observed in enriched pathways may be attributable to genes that were differentially expressed in a statistically significant manner. However, others may be the result of a less significant over/underexpression of a group of genes involved in the pathway, highlighting the importance of pathway enrichment as well as differential expression. Pathway scores generated by NanoString software provided more information about how each pathway was expressed in each individual, thus revealing an association between multiple clinical metabolism-related parameters and inflammation markers. Remarkably, correlation analyses revealed that systemic inflammation markers were generally positively associated with core energy-producing pathways, while showing negative correlations with anabolic processes. This pattern suggests that, during systemic inflammation, cellular activity shifts toward immediate energy generation at the expense of growth, biosynthesis, and long-term metabolic control, representing a catabolic, immunometabolic state.

An intricate interplay between immune and metabolic pathways takes place in kidney diseases [25]. Our exploratory analysis of PBMC-enriched ligands and receptors point to several immune–kidney signaling axes as candidates for further investigation in the context of DKD. Notably, the expression of receptors corresponding to PBMC-enriched ligands was altered in kidney cells following co-culture with PBMCs from patients with type 2 diabetes and DKD, thus supporting the presence of active intercellular communication. *SDC1*, a receptor of CCL5 involved in leukocyte recruitment, matrix remodeling and resolution of inflammation [26], was increased in kidney cells in co-culture with PBMCs from patients with type 2 diabetes with and without DKD, which is consistent with the enhanced proinflammatory activity of PBMCs in these pathologies. Similarly, DPP4, IL6st, and IL6R receptors were also upregulated in type 2 diabetes and DKD co-culture conditions. Interestingly, their ligands (*PTPRC, PIK3CA* for *DPP4*, *IL6* for *IL6st*, and *IL6R*) were downregulated in type 2 diabetes vs controls and DKD vs controls, suggesting the existence of compensatory mechanisms rather than simple linear ligand-driven activation. DPP4 activity is increased in DKD kidney models and is involved in fibrosis [27], while IL6 exerts a dual pro- and anti-inflammatory role [28], pointing to a critical role of both molecules in DKD progression. In short, our findings indicate that PBMCs from patients with type 2 diabetes and DKD can modulate renal gene expression.

The first limitation of this study is the relatively small sample size employed for the Nanostring analysis. However, it does not affect the transferability of our findings, as it renders a statistical power of 80.6% to detect differentially expressed genes at a minimum log2FC = 1 and a false discovery rate of 5%, thus meeting the conventional 80% standard. Furthermore, this sample size is consistent with published recommendations for transcriptomic studies, where n≈12 per group has been shown to represent a reasonable balance between statistical robustness and feasibility [29, 30]. We acknowledge that false negatives may have occurred, as genes with smaller effect sizes (log2FC < 1) may not have been detected due to the limited statistical power of the small sample. Nevertheless, the genes identified as significantly altered represent robust and reliable signals. To back up our conclusions regarding *CPT1A*, we used RT-qPCR to assess its expression in a larger cohort of 95 subjects, which confirmed similar upregulation in the type 2 diabetes and DKD groups. However, ROC and AUC analyses indicated a limited discriminatory performance of *CPT1A* in the RT-qPCR cohort, which points to it being a candidate for further analysis rather than a definitive biomarker.

Another limitation is that our control group was younger than our type 2 diabetes and DKD groups, and the type 2 diabetes had higher BMI than the other two groups. We acknowledge the difficulty of discriminating between specific effects of BMI and those of diabetes given that 82% of our type 2 diabetes patients had a BMI ≥ 25 kg/m^2^, which is representative of that population [31]. Taking into account that type 2 diabetes prevalence increases with age and obesity, recruiting age-matched healthy controls is challenging, as older individuals often present age-related metabolic and immune alterations. Consequently, many case–control studies in the literature include control groups that differ in age from type 2 diabetes patients [32–34]. Although our statistical analyses were adjusted for age and BMI, these two variables continue to be potential confounders that could have specific effects on metabolism-related genes. Despite the sample size precluding adjustment for pharmacological treatment in the statistical model, a Fisher's exact test revealed no significant differences in the distribution of any of the 11 drug classes examined between the type 2 diabetes and DKD groups (*p* > 0.05), suggesting that differential medication use is unlikely to account for the differences in gene expression observed between these groups.

In short, we describe differences in the transcriptomic profiles of PBMCs from overweight patients and those with type 2 diabetes and DKD with respect to healthy subjects and demonstrate that these differences are associated with clinical parameters and inflammation. Indeed, our correlation analysis suggests that, during systemic inflammation, cells prioritize immediate energy production over growth, biosynthesis, and long-term metabolic regulation, reflecting a shift toward a catabolic, immunometabolic state.

## Materials and methods

### Subjects and assessment of clinical parameters

Patients with type 2 diabetes (group D; *n* = 12) and patients with type 2 diabetes and DKD (group DKD; *n* = 12) were recruited from the Endocrinology and Nutrition and Nephrology Services of University Hospital Doctor Peset (Valencia, Spain) during routine clinical appointment and follow-up visits. Control volunteers were hospital workers’ family members, friends and acquaintances (group C; *n* = 12). Type 2 diabetes diagnosis was established following ADA guidelines [35], while DKD diagnosis was based on KDIGO criteria [6]. Eligibility criteria included: 18–70 years old, BMI < 40 kg/m^2^, and a known evolution of the disease of over 5 years. Self-reported sex and ethnicity data were collected. Other moderate-to-severe inflammatory, malignant, organic, haematological, or infectious diseases were exclusion criteria. The study protocol was approved by the Ethics Committee for Clinical Research with Medicines of University Hospital Dr. Peset (CEIm; ID:100.22) and complied with the principles of the Declaration of Helsinki. Participants gave their written, informed consent. Blood biochemical determinations were performed by the hospital’s clinical analysis service [9].

### Leukocyte isolation

Blood samples were drawn between 8:00 and 9:00 under fasting conditions using EDTA-coated tubes. PBMCs and neutrophils were isolated using MACSprep kits (130–115–169; Miltenyi Biotec, Germany) and MACSxpress Separator (130–115–319; Miltenyi Biotec, Germany), respectively. PBMCs were resuspended in 50µL of RNAlater stabilization solution (AM7020; Thermo Fisher, USA) and preserved at −80ºC until RNA extraction, while neutrophils were immediately used for the leukocyte-endothelium assays.

### Flow cytometry

Whole blood was lysed to remove erythrocytes (130-094-183; Miltenyi Biotec, Germany) and leukocytes were marked with APC-CD45 antibody (555485 BD biosciences, NJ, USA) for 20 min in the dark. PBMCs were gated using CD45 and side scatter signals. Separately, mitochondrial membrane potential was evaluated with tetramethyl rhodamine methyl ester (TMRM, 3 µM, ThermoFisher, 11550796, Waltham, MA USA), total ROS production with 2',7'-dichlorodihydrofluorescein diacetate (DCFH-DA, 5 µM, ThermoFisher, 11500146, Waltham, MA USA), mitochondrial ROS production with MitoSox Red (3 µM, ThermoFisher, 11579096, Waltham, MA USA) and mitochondrial mass with Mitotracker Green (6.6 nM, ThermoFisher, M7514, Waltham, MA USA). All samples were incubated for 10 min in the dark at room temperature before measurement. Fluorescence was measured in a C6 Accuri cytometer (BD Biosciences, NJ, USA) with a blue laser (488 nm) and FL3 filter (585/40 nm), and Ex/Em = 550/575 for TMRM. 10,000 cells were analysed in each experiment.

### Neutrophil-endothelium interaction assays

A confluent monolayer of HUVEC/TERT2 cells (CRL-4053; ATCC, VA, USA) was cultured in 35 ×10 mm culture dishes (430,165; Corning, MS, USA) with Vascular Cell Basal Medium (PCS-100–030; ATCC, VA, USA) supplemented with the Endothelial Cell Growth Kit VEGF (PCS-100–041; ATCC, VA, USA) and 0.5% Penicillin/Streptomycin (P/S) vol./vol. Neutrophil-endothelium interactions were evaluated using a parallel flow chamber within a dynamic adhesion system, as previously described [36]. Rolling velocity (µm/s), rolling flux (cells/min), and adhesion (cells/mm^2^) parameters were calculated using Tracker (free software from physlets.org/tracker/).

### RNA extraction and quantification

RNA was extracted from PBMCs (2.5 × 10^6^ cells) using Ribospin RNA extraction kits (304-150; GeneAll, Korea). RNA quantity and quality were evaluated by Nanodrop 2000 (ThermoFisher) and Bioanalyzer 2100 (Agilent Technologies, Santa Clara, CA, USA).

### mRNA expression analysis

mRNA expression was measured in 400 ng of RNA with the nCounter® Human Metabolic Pathway Panel from NanoString (XT-CSO-HMP1-12, NanoString; Seattle, WA) at Citogen (Zaragoza, Spain) and included 748 genes across 36 annotated pathways (Table S6, https://nanostring.com/). Raw data was processed using nSolver® Analysis Software (nanoString Technologies, version 4.0). In brief, background signal was removed by background thresholding (mean of negative control signal ±2SD), and positive control probes A–E were included for calculating the normalization factors as the ratios between the average of the geometric means and the geometric mean of each sample. Positive control F was excluded for having similar counts to negative control samples. Gene expression counts were then normalized with the geometric mean of 10 housekeeping genes (*MRPS5, NRDW2, COG7, SAP130, AGK, TLK2, SDHA, FCF1, DNAJC14, TBP*). Housekeeping genes with counts lower than 100 and a coefficient of variation (CV%) higher than 35 were excluded from this analysis. Two-fold ratios between all groups were calculated. Analyses were adjusted for Cartridge ID, as it could have acted as a confounder (Fig. S6).

### RT-qPCR validation

RNA was converted to cDNA using RevertAid first-strand cDNA synthesis kit (K1622; Thermo Fisher). The expression of *CPT1A* and *18S* was detected by amplification in a 7500 fast real-time PCR system thermocycler (Thermo Fisher) using 5 ng cDNA and FastStart Universal SYBR Green (491385001; Sigma-Aldrich, USA). Primer sequences were designed using NCBI BlastN and are shown in Table S7. Results were normalised to the expression of the housekeeping gene *18S* RNA and are expressed as ΔΔ*C*_t_ values. Samples were analysed in duplicate and ‘no template’ controls were added during the RT-qPCR process.

### Palmitate oxidation stress test

Cellular oxygen consumption rate (OCR) was measured using an Agilent Seahorse XF HS Mini Analyzer (Agilent Technologies) in PBMCs at a density (3 × 10^5^ cells/well). OCR was assessed using a protocol based on the XF Palmitate Oxidation Stress Test. Cells were incubated in XF DMEM Seahorse medium supplemented with 2 mM glucose in a non-CO₂ incubator at 37ºC and treated with either bovine-serum albumin (BSA) or BSA-conjugated palmitate. Basal OCR and responses to sequential injections of etomoxir, oligomycin, BAM15, and rotenone/antimycin A were recorded according to the manufacturer’s guidelines.

### HEK293 cell culture and coculture conditions

Human embryonic kidney-derived HEK-293.2sus (ATCC® CRL-1573.3™) cells were seeded in 6-well plates (1 × 10^4^ cells/cm^2^, Multiwell™, 353046) in DMEM supplemented with 10% fetal bovine serum (FBS) and 1% P/S and maintained at 37 °C in 5% CO₂ until confluence. Authentication of the cell lines was performed using short tandem repeat (STR) analysis and matched the reference profiles provided by ATCC. The authentication report is available in the Electronic Supplementary Material (ESM). Mycoplasma contamination testing was routinely performed and tested negative.

PBMCs isolated from patients (as described in “Leukocyte extraction and isolation” section) were added to HEK293 monolayers (1 × 10⁶cells/mL) in RPMI-1640 containing 10% FBS and 1% P/S. Cocultures were maintained for 24 h at 37 °C in 5% CO₂. After 24 h, PBMCs were removed, and adherent HEK293 cells were washed with phosphate-buffered saline and lysed with 600µL RLT buffer (Qiagen, 74106) supplemented with 1% (v/v) β-mercaptoethanol. Total RNA was purified using the RNeasy® Mini Kit (Qiagen, 74106) and cDNA synthesis and RT-qPCR was performed as detailed in the “ RT-qPCR validation” section. The primer sequences were designed using NCBI BlastN and are shown in Table S7.

### Statistical analysis

Transcriptomic data were analyzed using nSolver and R (versions listed in Table S8; details in the ESM). Participant characteristics and correlations were analyzed in GraphPad Prism 9.0.2 (www.graphpad.com) and SPSS (SPSS Statistics Inc) using ANOVA or Kruskal–Wallis tests, as appropriate. Sex differences were assessed by chi-square test. A univariate general linear model was used to adjust for age and BMI. Correlations were evaluated using Spearman coefficients, with multiple-testing correction by the Benjamini–Hochberg method. Statistical significance was set at *p* < 0.05.

## Supplementary Information

Supplementary Material 1.

## Data Availability

The transcriptomic data generated and analyzed in this study are publicly available in the GEO repository under accession number GSE316126 (https://www.ncbi.nlm.nih.gov/geo/query/acc.cgi?acc=GSE316126) and in the Zenodo repository (DOI: 10.5281/zenodo.17408527). Complete results of the differential gene expression analyses, functional enrichment data and all supplementary tables, can be accessed in the Zenodo repository. Datasets GSE142153, GSE154881, and GSE185011 are also available in the GEO repository (https://www.ncbi.nlm.nih.gov/geo/query/acc.cgi?acc=GSE142153, https://www.ncbi.nlm.nih.gov/geo/query/acc.cgi?acc=GSE154881, and https://www.ncbi.nlm.nih.gov/geo/query/acc.cgi?acc=GSE185011, respectively).

## Abbreviations

ARID2: AT-Rich Interaction Domain 2
ASH1L: ASH1 Like Histone Lysine Methyltransferase
BMI: Body-mass index
BSA: Bovine serum albumin
CCL5: C-C Motif Chemokine Ligand
CKD: Chronic kidney disease
CPT1A: Carnitine palmitoyl transferase 1A
CV%: Coefficient of variation
DCFH-DA: 2',7'-Dichlorodihydrofluorescein diacetate
DEGs: Differentially expressed genes
DKD: Diabetic kidney disease
DPP4: Dipeptidyl Peptidase 4
ESRD: End-stage renal disease
FBS: Fetal bovine serum
GBA: Glucosylceramidase Beta 1
GO: Gene ontology
GEO: Gene Expression Omnibus
hsCRP: High-sensitivity C-reactive protein
HbA1c: Glycated haemoglobin
HOMA-IR: Homeostatic Model Assessment for Insulin Resistance
HDL: High density lipoprotein
HLA-DQA1: Major Histocompatibility Complex Class II DQ Alpha 1
HUVEC: Human umbilical vascular endothelial cells
ICOS: Inducible T Cell Costimulator
IDO1: Indoleamine 2,3-Dioxygenase 1
IL6st and IL6R: IL6 receptors
INFAR1/2: Interferon Alpha and Beta Receptor Subunit 1 and 2
IRF4: Interferon Regulatory Factor 4
KEGG: Kyoto Encyclopedia of Genes and Genomes
LDL: Low density lipoprotein
MLR: Monocyte-to-lymphocyte ratio
NLR: Neutrophil-to-lymphocyte ratio
OCR: Oxygen consumption rate
PBMCs: Peripheral blood mononuclear cells
PIK3CA: Phosphatidylinositol-4,5-Bisphosphate 3-Kinase Catalytic Subunit Alpha
PLR: Platelet-to-lymphocyte ratio
PPP: Pentose phosphate pathway
PTPRC: Protein Tyrosine Phosphatase Receptor Type C
P/S: Penicillin/Streptomycin
ROS: Reactive oxygen species
RT-qPCR: Quantitative reverse transcription polymerase chain reaction
SDC1/4: Syndecan 1 or 4
SII: Systemic Inflammatory Index
SLC7A11: Solute Carrier Family 7 Member 11
SMAD3: SMAD Family Member 3
TET2: Tet Methylcytosine Dioxygenase 2
TMRM: Tetramethyl rhodamine methyl ester
