# Supplementary material for: Distinct immunometabolic signatures in peripheral blood mononuclear cells are linked to systemic inflammation in type 2 diabetes and diabetic kidney disease
Source: Mol Biomed. 2026 Aug 8;7:133. doi: 10.1186/s43556-026-00523-3 (PMC13451171; doi:10.1186/s43556-026-00523-3)
Supplement: Supplementary file 1 [file 43556_2026_523_MOESM1_ESM.zip › ESM_ESM.pdf]

ELECTRONIC SUPPLEMENTARY MATERIAL

Distinct immunometabolic signatures in peripheral blood mononuclear cells are linked to systemic inflammation in type 2 diabetes and diabetic kidney disease

Clara Luna-Marco[1#], José F. Català-Senent[2#], Julia Cacace[3], Alberto Hermo-Argibay[3], Omar A. Hernández-López[3], María Pelechá-Salvador[3], Jonay Pantoja[4], Asunción Sancho[4], Jose Vila[1], Carlos Morillas[3], Celia Bañuls[3], Milagros Rocha[3], Víctor M. Víctor[1,3]*, Susana Rovira-Llopis[3]*

[1]Department of Physiology, University of Valencia, INCLIVA (Biomedical Research Institute Valencia), Valencia, Spain

[2]Microbiology and Ecology Department, University of Valencia, Valencia, Spain

[3]Service of Endocrinology and Nutrition, University Hospital Doctor Peset, Foundation for the Promotion of Health and Biomedical Research in the Valencian Region (FISABIO), Valencia, Spain

[4]Service of Nephrology, University Hospital Doctor Peset, Foundation for the Promotion of Health and Biomedical Research in the Valencian Region (FISABIO), MITOMETS, Valencia, Spain

[#] These authors have contributed equally

*Corresponding authors: susana.rovira@uv.es, victor.victor@uv.es

Luna-Marco, Clara ORCID: 0000-0003-1704-7051 cluna@incliva.es

Català-Senent, José Francisco ORCID: 0000-0002-5892-7437 jfcatala@gmx.com

Cacace, Julia ORCID: 0009-0000-6948-200X julia.cacace@fisabio.es

Hermo-Argibay, Alberto ORCID: 0000-0001-5760-0363 alberto.hermo@fisabio.es

Hernández-López, Omar A. ORCID: 0009-0000-0289-2485 omar.hernandez@fisabio.es

Pelechá-Salvador, María ORCID: 0000-0002-2141-6422 maria.pelecha@fisabio.es

Pantoja, Jonay ORCID: 0000-0003-3458-5425 jonay.nefro@gmail.com

Sancho, Asunción ORCID: 0000-0002-4170-9003 sancho_asucal@gva.es

Vila, Jose ORCID: 0000-0001-8589-9307 jose.m.salinas@uv.es

Morillas, Carlos ORCID: 0000-0002-3745-4423 carlos.morillas@uv.es

Bañuls, Celia ORCID: 0000-0001-8077-7642 celia.banuls@fisabio.es

Rocha, Milagros ORCID: 0000-0003-2923-6546 milagros.rocha@fisabio.es

Víctor, Víctor M. ORCID: 0000-0002-3027-3945 victor.victor@uv.es

Rovira-Llopis, Susana ORCID: 0000-0002-8476-5128 susana.rovira@uv.es

Supplementary Tables:

Supplementary table 1. Treatment of patients expressed in percentage of patients per group (D= Type 2 diabetes; DKD= Type 2 diabetes and diabetic kidney disease).

	% of patients		Chi^2
**Drug**	**D**	**DKD**	***p* value**
Antihypertensive drugs	50.0	41.7	ns
Metformin	83.3	75.0	ns
DPP4 inhibitors	16.67	50.0	ns
Insulin	58.3	58.3	ns
Statins	75.0	100	ns
GLP1-RA	41.7	33.3	ns
iSGLT2	100	91.7	ns

Supplementary table 2. Number of patients per category according to KDIGO guidelines (C= control, D= Type 2 diabetes; DKD= Type 2 diabetes and diabetic kidney disease).

	Albumin/creatin ratio (mg/g)		
**Glomerular filtrate (ml/min/1.23m²)**	**A1 (<30)**	**A2 (30-300)**	**A3 (>300)**
**G1 (≥90)**	9 C<br>8 D	7 DKD	0
**G2 (60-89)**	3 C<br>4 D	3 DKD	0
**G3a (45-59)**	1 DKD	0	0
**G3b (30-44)**	0	1 DKD	0
**G4 (15-29)**	0	0	0
**G5 (<15)**	0	0	0

Supplementary table 3. Characteristics of the participants for RT-qPCR validation.

	C	D	DKD	*p* value	Age & BMI adjusted *p*-value
n	33	32	30	-	-
Sex (% women)	66,67	37,5	66,67	ns	-
Age (years)	40±13	57±10***	64±9***	<0.001	-
BMI (kg/m^2)	23.1±2.9	30.6±4.5***	29.6±6.3	<0.001	-
HbA1c-DCCT%	5.25 (5.00-5.38)	6.68 (6.00-7.65)***	6.72 (6.30-7.55)***	<0.001	<0.001
Glucose (mg/dL)	86.5 (82.0-96.0)	118.8 (99.5-144.0)***	143.0 (121.6-183.5)***	<0.001	<0.001
Basal Insulin (uUI/mL)	6.50 (5.80-8.25)	10.80 (5.88-16.49)**	11.80 (8.20-20.70)***#	<0.001	<0.01
HOMA-IR	1.39 (1.20-1.74)	3.00 (2.08-4.05)***	4.96 (3.33-7.12)***	<0.001	<0.01
Cholesterol (mg/dL)	181 (164-213)	161 (140-182)*	154 (136-206)	<0.05	<0.05
HDL (mg/dL)	62.9 (45.8-69.0)	46.8 (43.0-53.0)**	44.5 (34.0-56.5)**	<0.001	ns
LDL (mg/dL)	108.0 (90.5-128.2)	87.0 (66.0-101.0)**	81.5 (60.5-118.5)	<0.01	<0.01
Triglycerides (mg/dL)	64.5 (51.2-108.6)	104.0 (84.4-145.4)**	156.0 (94.8-225.5)***#	<0.001	<0.01
Glomerular Filtration Rate (ml/min/1.73m^2)	101.5±11.5	93.2±13.7*	74.0±27.5***###	<0.001	ns
Albumin/creatinine ratio (mg/g)	4.90 (4.00-6.84)	9.39 (6.20-14.80)***	56.57 (37.00-138.27)***#	<0.001	<0.001
C-Reactive Protein (mg/L)	1.17 (0.53-2.15)	1.93 (0.98-4.21)*	3.82 (0.90-6.98)	<0.05	ns
Plasmatic Homocystein (µmol/L)	9.40 (8.10-12.00)	12.20 (8.90-13.00)	13.40 (10.08-22.40)**	<0.05	ns
Leukocytes (x10^9/L)	6.10 (5.40-7.20)	7.05 (6.15-8.05)**	8.70 (7.05-9.65)	<0.01	<0.01
Neutrophils (x10^9/L)	3.60 (2.90-4.30)	3.75 (3.18-4.63)*	6.00 (4.63-6.53)	<0.001	<0.001
Lymphocytes (x10^9/L)	1.90(1.60-2.20)	2.30 (1.98-2.80)	1.95 (1.52-2.38)	ns	ns
Monocytes (x10^9/L)	0.40 (0.40-0.50)	0.50 (0.40-0.6)	0.50 (0.50-0.63)	<0.05	ns
Eosinophils (x10^9/L)	0.20 (0.10-0.30)	0.20 (0.10-0.33)	0.14 (0.10-0.20)	ns	ns
Platelets (x10^9/L)	219 (201-283)	240 (217-265)	244 (186-287)	ns	ns
NLR	2.08±0.83	1.65±0.75	3.03±1.16	ns	<0.01
PLR	129±43	106±31	130±70	ns	ns
SII	499±238	394±166	739±416	ns	<0.05
MLR	0.24 (0.18-0.31)	0.21 (0.18-0.25)	0.30 (0.24-0.35)	ns	

C=control subjects, D=patients with type 2 diabetes, DKD= patients with type 2 diabetes and diabetic kidney disease, BMI=body-mass index, HbA1c= glycated haemoglobin, HOMA-IR= Homeostatic Model Assessment for Insulin Resistance, HDL: high density lipoprotein, LDL: low density lipoprotein NLR=neutrophil-to-lymphocyte ratio; PLR=platelet-to-lymphocyte ratio; SII=Systemic Inflammatory Index; MLR=monocyte-to-lymphocyte ratio. Normally distributed parameters are shown as mean ± SD, while non-normally distributed data are expressed as medians (25th-75th quantiles). Comparisons between groups were performed using an ANOVA and Fisher LSD or Kruskall-Wallis and Dunn tests for normally and non-normally distributed data, respectively. The $\chi 2$ test was used to compare proportions. The influence of age and BMI was tested and corrected using a general linear model. *p<0.05, **p<0.01, ***p<0.001 vs. C; #p<0.05, ##p<0.01, ###p<0.001 D vs. DKD.

Supplementary table 4. ROC and AUC analysis

Cohort	Comparison	AUC	95% CI	N cases	N controls	Power
NanoString	C vs. DKD	0.903	0.786–1.000	12	12	**0.991**
NanoString	C+D vs. DKD	0.785	0.628–0.941	12	24	**0.837**
NanoString	D vs. DKD	0.667	0.440–0.893	12	12	0.300
Validation	C vs. DKD	0.618	0.478–0.758	30	33	0.383
Validation	C+D vs. DKD	0.559	0.431–0.687	30	65	0.156
Validation	D vs. DKD	0.502	0.355–0.649	30	32	0.027

C=control subjects, D=patients with type 2 diabetes, DKD= patients with type 2 diabetes and diabetic kidney disease. AUC= area under the curve, CI= confidence interval.

Supplementary table 5. Characteristics of participants for functional validation cohort and co-culture with HEK293 cells

	C	D	DKD	*p* value	Age adjusted *p*-value
n	4	4	3	-	-
Sex (% women)	50	50	0	-	-
Age (years)	38±14	59±5*	67±4**	<0.01	-
BMI (kg/m^2)	25.1 (24.4-30.0)	28.5 (26.6-33.9)	27.3 (26.3-28.2)	ns	ns
HbA1c-DCCT%	4.65 (4.62-4.81)	8.10 (6.40-8.13)	7.48 (7.42-7.50)	ns	ns
Glucose (mg/dL)	84.0±2.0	101.7±28.7	149.3±16.0**	<0.05	ns
Basal Insulin (uUI/mL)	5.77±1.60	10.07±11.19	10.80±76.99	ns	ns
HOMA-IR	1.19±0.31	2.28±2.52	4.10±3.03	ns	ns
Cholesterol (mg/dL)	190±7	124±23	143±54	ns	ns
HDL (mg/dL)	51.3±10.7	49.0±1.0	47.0±12.1	ns	ns
LDL (mg/dL)	124 (115-127)	50.0 (49.0-67.5)	49.0 (48.0-85.0)	ns	ns
Triglycerides (mg/dL)	96.0±16.5	71.7±20.6	120.3±2.5	ns	ns
Glomerular Filtration Rate (ml/min/1.73m^2)	95.3±17.5	98.3±12.5	71.3±28.3	ns	ns
Albumin/creatinine ratio (mg/g)	4.00 (4-20.14)	22.8 (16.9-26.4)	106.0 (66.5-175.0)	ns	ns
C-Reactive Protein (mg/L)	2.00 (1.00-2.00)	1.00 (1.00-3.00)	2.00 (2.00-3.50)	ns	ns
Plasmatic Homocystein (µmol/L)	10.17±11.20	9.83±3.89	13.2±2.220	ns	ns
Leukocytes (x10^9/L)	5.03±0.81	6.03±0.67	6.80±0.56	<0.05	ns
Neutrophils (x10^9/L)	2.70 (2.60-2.80)	3.50 (3.30-3.50)	4.00 (3.85-4.15)*	<0.05	ns
Lymphocytes (x10^9/L)	1.83±0.57	1.97±0.65	1.87±0.32	ns	ns
Monocytes (x10^9/L)	0.30 (0.30-0.35)	0.40 (0.40-0.45)	0.60 (0.45-0.75)	ns	ns
Eosinophils (x10^9/L)	0.00 (0.00-0.05)	0.00 (0.00-0.05)	0.10 (0.05-0.15)	ns	ns

Platelets ($x10^9$/L)	255±94	288±100	265±82	ns	ns
NLR	1.57±0.46	1.87±0.76	2.18±0.43	ns	ns
PLR	137±17	170±120	150±16	ns	ns
SII	372±75	583±432	594±277	ns	ns
MLR	0.19±0.06	0.23±0.06	0.35±0.23	ns	ns

C=control subjects, D=patients with type 2 diabetes, DKD= patients with type 2 diabetes and diabetic kidney disease, BMI=body-mass index, HbA1c= glycated haemoglobin, HOMA-IR= Homeostatic Model Assessment for Insulin Resistance, HDL: high density lipoprotein, LDL: low density lipoprotein NLR=neutrophil-to-lymphocyte ratio; PLR=platelet-to-lymphocyte ratio; SII=Systemic Inflammatory Index; MLR=monocyte-to-lymphocyte ratio. Normally distributed parameters are shown as mean ± SD, while non-normally distributed data are expressed as medians (25th-75th quantiles). Comparisons between groups were performed using an ANOVA and Fisher LSD or Kruskall-Wallis and Dunn tests for normally and non-normally distributed data, respectively. The $\chi 2$ test was used to compare proportions. The influence of age and BMI was tested and corrected using a general linear model. *p<0.05, **p<0.01 vs. C.

Supplementary table 6. nCounter® Metabolic Pathways Panel Gene List (LBL-10725-01) (Excel File)

Supplementary table 7. Primer sequences

**Gene**	**Forward sequence (5’-> 3’)**	**Reverse Sequence (5’-> 3’)**	**Amplicon size (bp)**
CPT1A	GATCCTGGACAATACCTCGGAG	CTCCACAGCATCAAGAGACTGC	142
18S	ACCCGTTGAACCCCATTCGTGA	GCCTCACTAAACCATCCAATCGG	159
SDC1	TCCTGGACAGGAAAGAGGTGCT	TGTTTCGGCTCCTCCAAGGAGT	142
SDC4	GAGTGAGGATGTGTCCAACAAGG	GGTACATGAGCAGTAGGATCAGG	149
DPP4	AAAGGCACCTGGGAAGTCATCG	CAGCTCACAACTGAGGCATGTC	153
IFNAR1	CGCCTGTGATCCAGGATTATCC	TGGTGTGTGCTCTGGCTTTCAC	156
IFNAR2	ACCGTCCTAGAAGGATTCAGCG	CCAACAATCTCAAACTCTGGTGG	107
IL6st	CACCCTGTATCACAGACTGGCA	TTCAGGGCTTCCTGGTCCATCA	134
IL6R	GACTGTGCACTTGCTGGTGGAT	ACTTCCTCACCAAGAGCACAGC	146

Supplementary table 8. R versions.

**R software / package**	**Version**
R	4.5.1
ComplexHeatmap	2.24.0
EnhancedVolcano	1.26.0
ggrepel	0.9.6
mdgsa	0.99.2
org.Hs.eg.db	3.21.0
AnnotationDbi	1.70.0
Iranges	2.42.0
S4Vectors	0.46.0
Biobase	2.68.0
BiocGenerics	0.54.0
readxl	1.4.5

writexl	1.5.4
tidyverse	2.0.0
ggplot2	3.5.2
patchwork	1.3.0
factoextra	1.0.7
corrr	0.4.4
RnaSeqSampleSize	2.20.0
pROC	1.19.0.1
KEGGREST	1.48.0

Supplementary Figures:

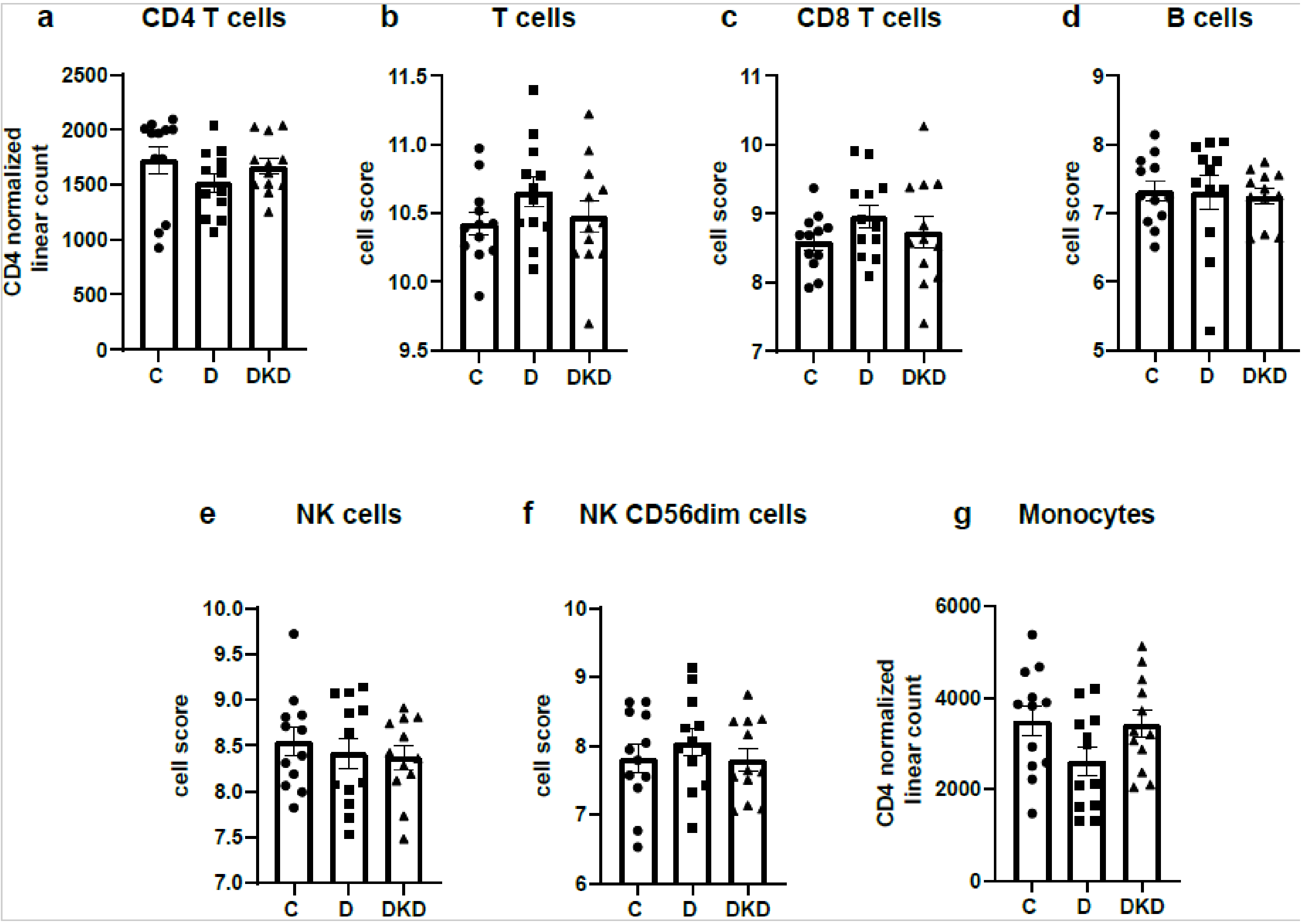

Supplementary figure 1. Analysis of expression levels of cell type–specific gene markers or cell type scores retrieved from Danaher et al. (https://doi.org/10.1186/s40425-017-0215-8) in the Nanostring dataset. C=control subjects, D=patients with type 2 diabetes, DKD= patients with type 2 diabetes and diabetic kidney disease.

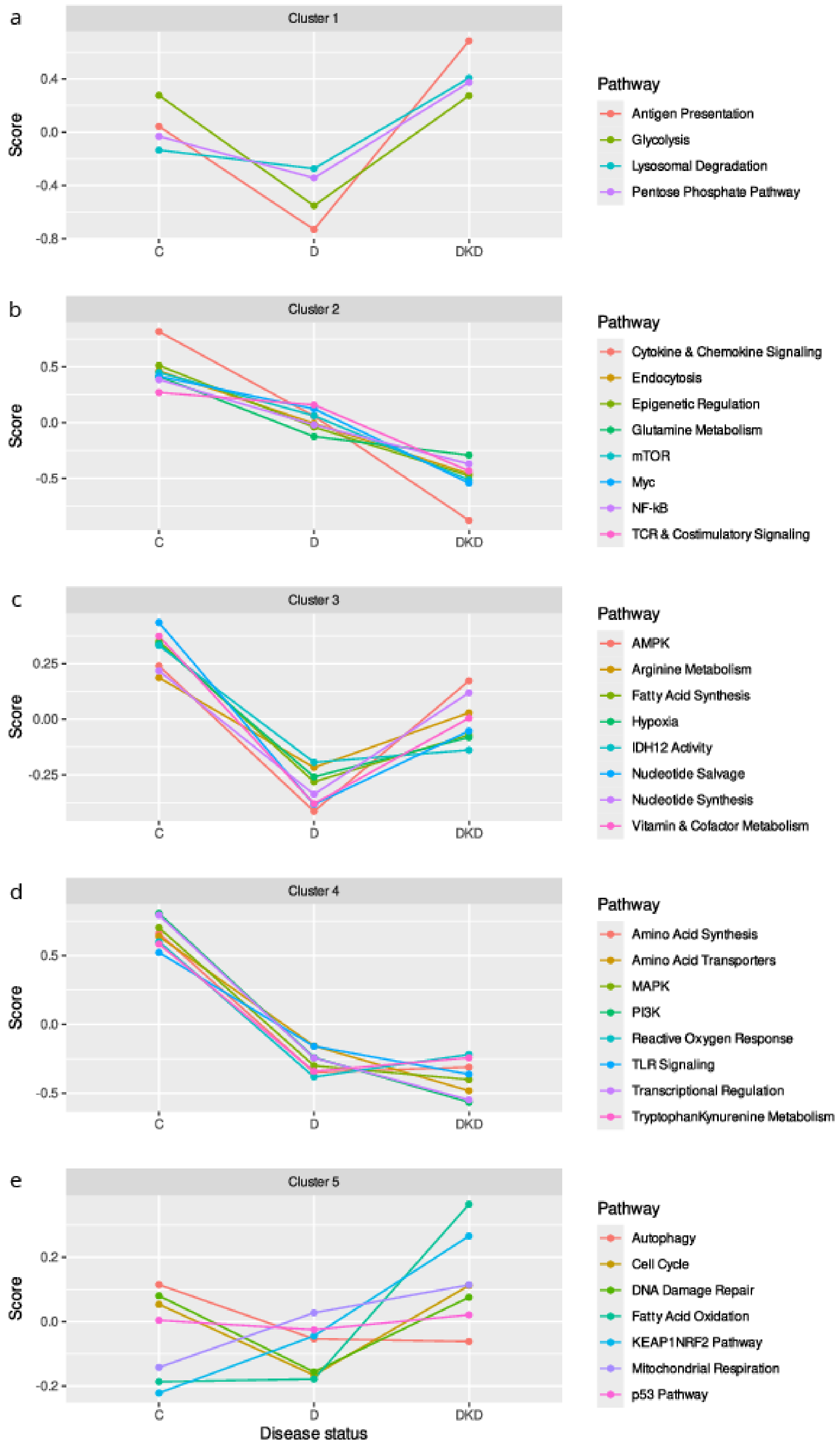

Supplementary figure 2. Patterns of expression from pathway scores when comparing controls (C), type 2 diabetes (D), and type 2 diabetes with diabetic kidney disease (DKD) patients. The points represent the average expression of each pathway. Pathways were grouped into 5 clusters using the k-means method.

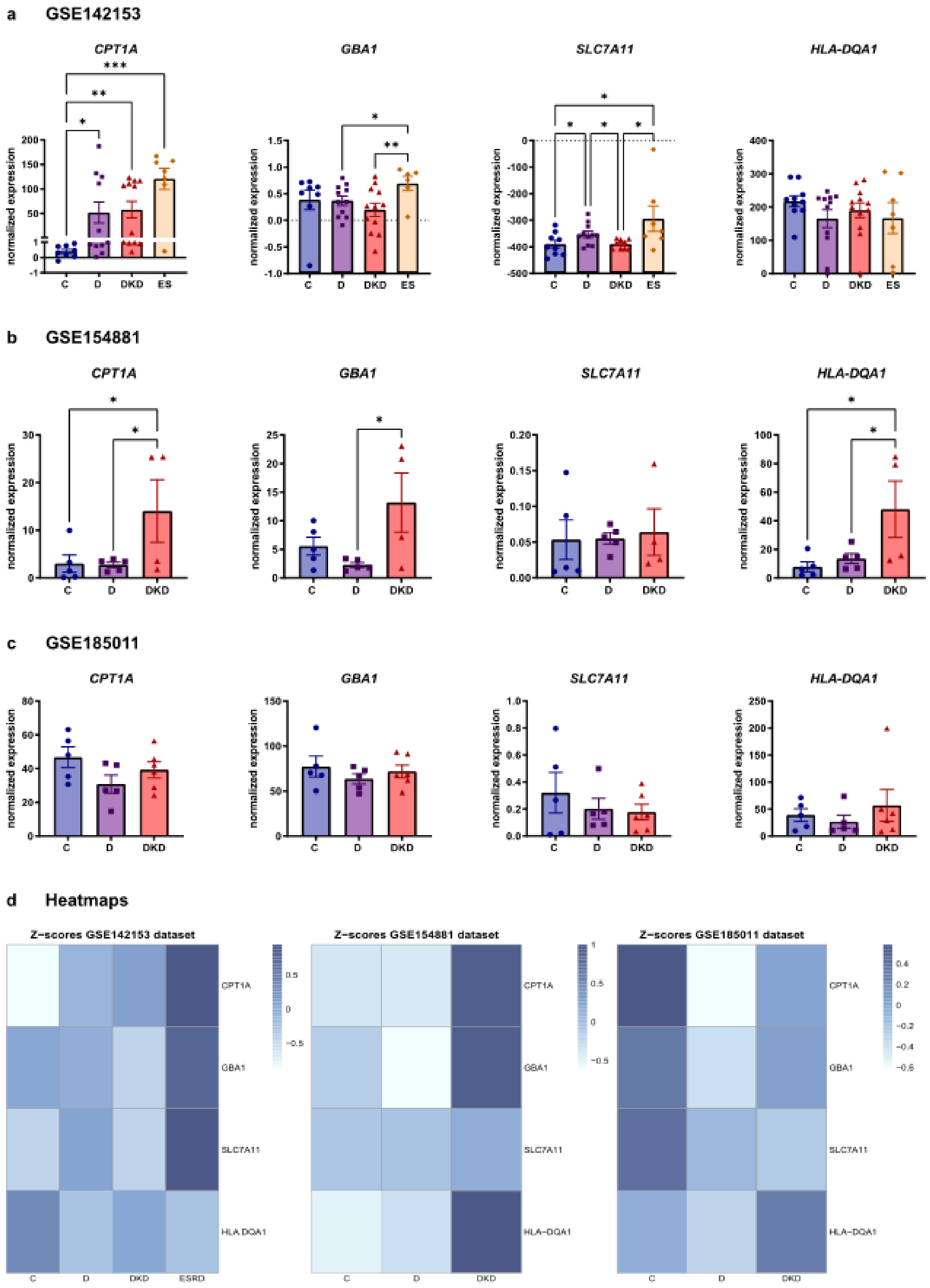

Supplementary figure 3. Database validation results for genes *CPT1A*, *GBA1*, *SLC7A11* and *HLA-DQA1* with databases GSE142153 (a), GSE154881 (b) and GSE185011 (c) and heatmap with Z-scores for each database (d). One way ANOVA or Kruskal Wallis tests, *p<0.05, **p>0.01, ***p<0.001. C: control, D: type 2 diabetes, DKD: diabetic kidney disease, ES: end-stage renal disease.

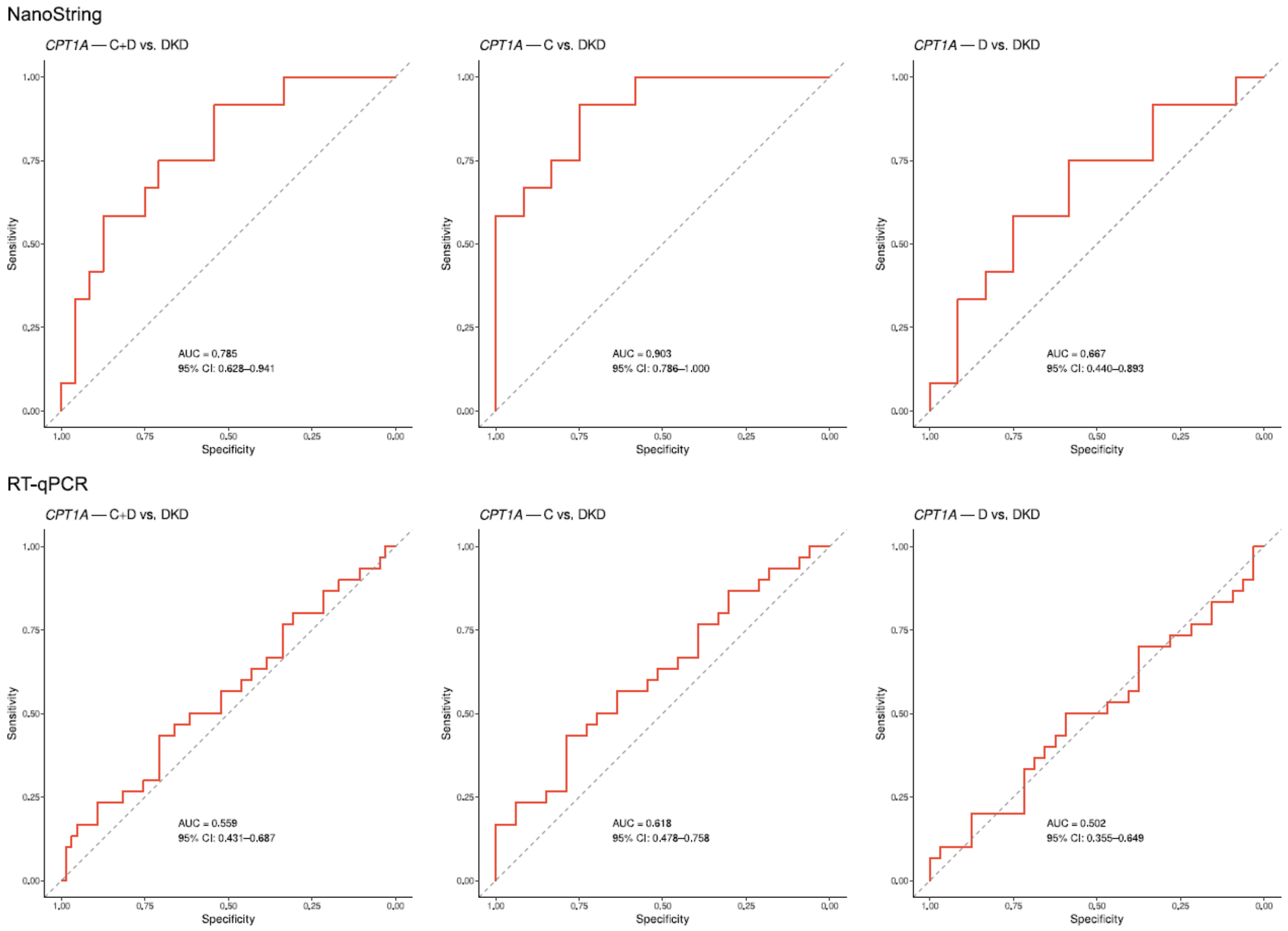

Supplementary figure 4. ROC curves and AUC analysis for CPT1A as potential biomarker in the Nanostring and qPCR cohorts. C=control subjects, D=patients with type 2 diabetes, DKD= patients with type 2 diabetes and diabetic kidney disease.

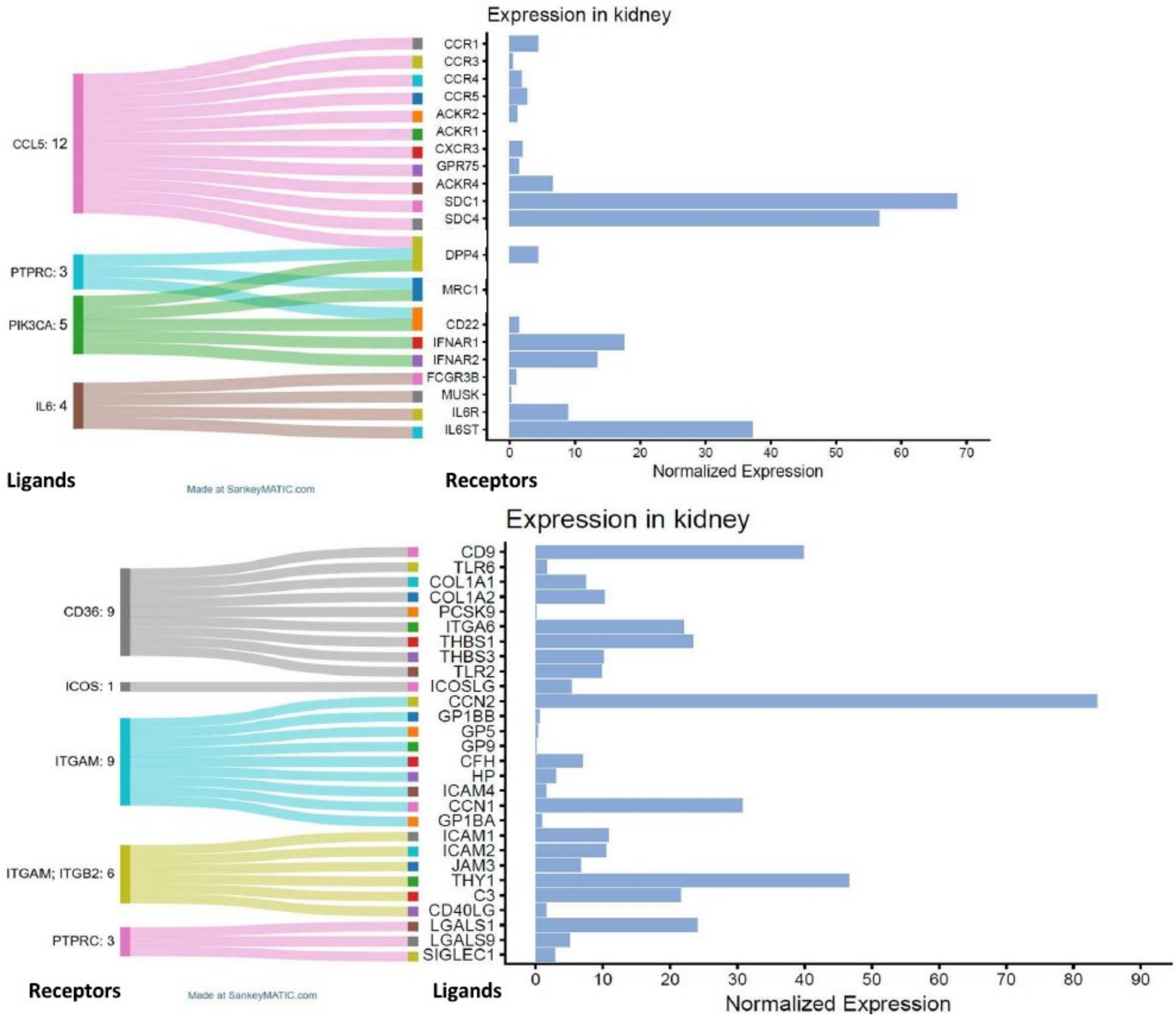

Supplementary figure 5. Expression of ligands and receptors in kidney of the DEGs from PBMCs. Data obtained from Cellinker platform (rna-society.org/cellinker/index.html).

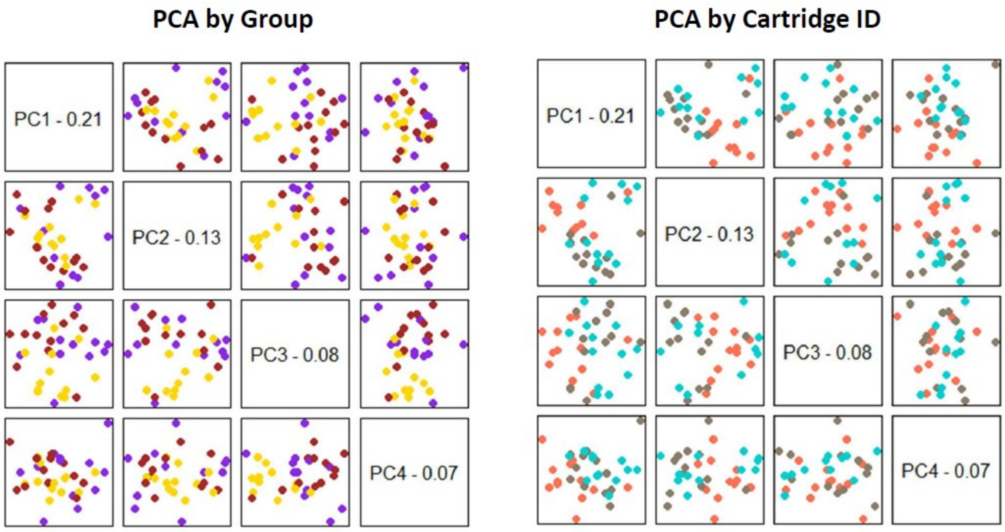

Supplementary figure 6. Data clustering by group and cartridge ID shown by PCA. Colors represent the three groups or cartridges, respectively.

Supplementary Material & Methods:

Bioinformatics Analysis

Raw data files (.RCC) were loaded into NanoString's nSolver Analysis Software (version 4.0.70), and each sample was annotated according to its experimental group and the cartridge it originated from. To minimize background noise, the Background Threshold option was utilized, setting the threshold to the mean counts of the negative control probes included in the assay. After this background correction, the data were normalized. First, a normalization factor was calculated using the geometric mean of the positive control probe counts to adjust for lane-to-lane technical variation and differences in assay efficiency. Next, a second normalization factor was computed from the geometric mean of a set of stably expressed reference (housekeeping) genes, which corrected for variations in RNA input amounts across the samples.

Quality control (QC) metrics were evaluated for each sample and cartridge within nSolver. These metrics included imaging QC, binding density, positive control linearity, and limit of detection. Samples that flagged any QC issues were reviewed and, if necessary, excluded from further analysis. The normalized count data were then exported for statistical analysis to assess differential gene expression between the experimental groups (D vs C, DKD vs C and DKD vs D) and to determine the pathway scores. Differentially expressed genes (DEGs) with Benjamini-Hochberg adjusted $p<0.05$ were considered statistically significant. Pathway scores were calculated as a summary of the expression of different genes associated with the same functional pathway. This parameter represents a general shift in the expression of genes participating in a particular pathway for each individual [1] and allowed us to correlate the pathway scores to clinical, anthropometric and biochemical parameters. Pathways were grouped into 5 clusters using the k-means method.

Gene enrichment analysis was performed for each of the three DGE comparisons using biological processes from the Gene Ontology (BP) [2] and pathways from the Kyoto Encyclopedia of Genes and Genomes (KEGG) [3] as functional annotations. First, genes were ranked based on their p-values and the sign of the statistic obtained from the differential gene expression analysis. Gene Set Analysis was then conducted utilizing the logistic regression model implemented in the mdgsa R package [4]. According to the guidelines provided by the authors of the R package, annotations containing more than 500 genes were filtered out as

they were deemed too general to be informative. Conversely, annotations with fewer than 10 genes were discarded because they were considered too small to be where considered to be too small to be properly fitted by the multivariate logistic model [4] . Finally, functions with a Benjamini-Hochberg adjusted p-value of less than 0.05 were deemed significant.

Sample size and statistical power were estimated using the RnaSeqSampleSize package in R (Developer S, Chung-I Li, Yan Guo, Quanhu Sheng, Yu Shyr (2025). *RnaSeqSampleSize: RnaSeqSampleSize*., package version 2.21.0, [5]), based on the empirical count dispersion distribution of the NanoString cohort. Dispersion parameters were estimated from raw count data across the three study groups (C, DKD, D; n=12 per group) using a negative binomial model, yielding a mean dispersion of 0.094 and a biological coefficient of variation (BCV) of 0.307. Power simulation (500 iterations) indicated that a sample size of n=12 per group achieves 80.6% statistical power to detect differentially expressed genes at a minimum fold change of 2.0 and a false discovery rate threshold of 5%, assuming 20 truly differential genes among the 547-gene panel. This meets the conventional 80% power threshold, supporting the adequacy of the study design. However, statistical power decreases substantially for fold changes below 2.0, and genes with smaller effect sizes may not be reliably detected within this sample size, representing an inherent limitation of the current study.

Publicly available mRNA expression datasets were retrieved from the Gene Expression Omnibus (GEO) database (accession numbers GSE142153, GSE154881 and GSE185011). Normalized expression data and Z-scores were used to compare the expression of the genes of interest among groups.

Volcano plots and correlation matrices were generated using the R packages EnhancedVolcano [6] and ComplexHeatmap [7], respectively. The versions of all R packages utilized in the bioinformatic analysis are detailed in Supplementary table 7.

ROC and AUC analysis

To assess the discriminative capacity of *CPT1A* expression as a biomarker for diabetic kidney disease (DKD), ROC (*Receiver Operating Characteristic*) curves were constructed for three pairwise comparisons in each cohort (NanoString and validation): controls + non-DKD diabetics (C+D) vs. DKD, controls (C) vs. DKD,

and non-DKD diabetics (D) vs. DKD. Empirical (non-smoothed) ROC curves were computed using the “roc” function from the pROC package [8], specifying the reference and positive group levels for each comparison. The area under the curve (AUC) was estimated with its 95% confidence interval using the DeLong method (“ci.auc”), and statistical power was assessed with the “power.roc.test” function. ROC curves were visualized using ggplot2 displaying the empirical curve (sensitivity vs. specificity), the no-discrimination reference line, and an annotation of the AUC with its 95% CI.

Ligand–receptor expression analysis

Ligand–receptor expression in kidney tissue corresponding to differentially expressed genes (DEGs) identified from peripheral blood mononuclear cells (PBMCs) was analyzed using the Cellinker platform [9, 10].

1. Tomfohr J, Lu J, Kepler TB (2005) Pathway level analysis of gene expression using singular value decomposition. BMC Bioinformatics 6(1):225. https://doi.org/10.1186/1471-2105-6-225
2. Ashburner M, Ball CA, Blake JA, et al (2000) Gene Ontology: tool for the unification of biology. Nat Genet 25(1):25–29. https://doi.org/10.1038/75556
3. Kanehisa M, Goto S (2000) KEGG: Kyoto Encyclopedia of Genes and Genomes. Nucleic Acids Res 28(1):27–30. https://doi.org/10.1093/nar/28.1.27
4. Montaner D, Dopazo J (2010) Multidimensional Gene Set Analysis of Genomic Data. PLOS ONE 5(4):e10348. https://doi.org/10.1371/journal.pone.0010348
5. RnaSeqSampleSize. In: Bioconductor. http://bioconductor.org/packages/RnaSeqSampleSize/.
6. EnhancedVolcano: publication-ready volcano plots with enhanced colouring and labeling. https://bioconductor.org/packages/release/bioc/vignettes/EnhancedVolcano/inst/doc/EnhancedVolcano.html.
7. Gu Z, Eils R, Schlesner M (2016) Complex heatmaps reveal patterns and correlations in multidimensional genomic data. Bioinformatics 32(18):2847–2849. https://doi.org/10.1093/bioinformatics/btw313
8. Robin X, Turck N, Hainard A, et al (2011) pROC: an open-source package for R and S+ to analyze and compare ROC curves. BMC Bioinformatics 12(1):77. https://doi.org/10.1186/1471-2105-12-77
9. Zhang Y, Liu T, Wang J, et al (2021) Cellinker: a platform of ligand–receptor interactions for intercellular communication analysis. Bioinformatics 37(14):2025–2032. https://doi.org/10.1093/bioinformatics/btab036
10. Home. https://rna-society.org/cellinker/index.html.

Cell Line Authentication Report by STR Profiling

1. Sample Information

Cell Line: HEK-293.2sus (ATCC® CRL-1573.3™)

Culture Conditions: Cells were cultured in T25 tissue culture flasks and were at passage 13 at the time of DNA extraction.

2. DNA Extraction

Genomic DNA was extracted using the QIAamp DNA Micro Kit (Qiagen) according to the manufacturer's instructions.

3. STR Profiling

Short Tandem Repeat (STR) profiling was performed using the ForenSeq Signature Plus Kit (Qiagen Cat no. / ID. V16000213) to obtain the genetic STR profile of the sample.

4. Reference Profile

The reference STR profile used for comparison was obtained from the quality control specifications provided by the American Type Culture Collection (ATCC) for the cell line HEK-293.2sus CRL-1573.3 (https://www.atcc.org/products/crl-1573.3).

5. Similarity Analysis

Similarity calculations between the obtained STR profile and the reference profile were performed using the Tanabe algorithm. The similarity score was calculated according to the following formula:

$$\text{Similarity} = \frac{2 \times N_{shared}}{N_{sample} + N_{reference}} \times 100$$

Where:

- N_{shared} = number of shared alleles between the sample and the reference profile.
- N_{sample} = total number of alleles detected in the sample.
- $N_{reference}$ = total number of alleles present in the reference profile.

The calculations are performed manually and in parallel using the CLASTR 1.5.0 web tool (https://www.cellosaurus.org/str-search/)

6. Genotype analysis and similarity calculation

The table below outlines the comprehensive STR marker analysis used for the cell line sample authentication:

Table 1: Comprehensive STR marker analysis used for cell line sample authentication. Discrepancies in heterozygosity at loci D21S11, D18S51, D7S820, and Penta_D indicate a partial loss of alleles or potential amplification bias in the sample relative to the reference.

STR Markers	Reference STR profiling	Sample STR profiling	Status / Observation
Amelogenin	X	X	Match
TH01	7,9.3	7,9.3	Match
D21S11	28,30.2	30.2	Loss of allele (28)
D18S51	17,18	17	Loss of allele (18)
Penta_E	7,15	7,15	Match
D5S818	8	8	Match
D13S317	12,14	12,14	Match
D7S820	11,12	11	Loss of allele (12)
D16S539	9,13	9,13	Match
CSF1PO	12	12	Match
Penta_D	9,10	9	Loss of allele (10)
D3S1358	15,17	15,17	Match
vWA	16,19	16,19	Match
D8S1179	12,14	12,14	Match
TPOX	11	11	Match
FGA	23	23	Match
D19S433	15,18	15,18	Match
D2S1338	19	19	Match

Tanabe algorithm calculation: (2 x 25 / 25 + 29) x 100 = 92,59%

7. Conclusion

The STR profile of the analyzed **cell line sample shows 92.59% similarity to the reference STR profile and therefore corresponds to the HEK-293.2sus CRL-1573.3** cell line. The profile shows loss of allelic heterozygosity in 4 of the 17 STRs analyzed. No clear tri-alellic patterns have been found, although the imbalances presented by some heterozygotes could suggest the presence of 3 alleles in some markers.

8. References

1. Korch CT et al. Human Cell Line Authentication: Standardization of Short Tandem Repeat (STR) Profiling, ASN-0002-2022. ATCC Standards Development Organization ASN-0002-2022. Manassas, VA, USA. https://webstore.ansi.org/standards/atcc/ansiatccasn00022022

2. Chen A, Chen J, Xuan Y, Zhao J, Ji C, Zhang S. Innovative use of forensic STRs for authentication of human cell lines stored over 34 years. *J Transl Genet Genom*. 2025;9:149-66 (https://dx.doi.org/10.20517/jtgg.2025.08).

València, May 18 2026

ALAN CODOÑER ALEJOS - NIF:29203055R

Firmado digitalmente por ALAN CODOÑER ALEJOS - NIF:29203055R
Fecha: 2026.05.18 14:15:51 +02'00'

Firmado por LUCIA MARTINEZ PRIEGO - NIF:***8913** el día 18/05/2026 con un certificado emitido por ACCV RSA1 CLIENTE

PhD Alan Codoñer Alejos
Genetic Technician

PhD Llúcia Martínez Priego
Scientific Director of FISABIO's Sequencing Service
